# Genetic Origin of *AHAS2* Genes in *Brassica* Allotetraploids and Association of Its Orthologs with Agronomic Traits in *B. napus*

**DOI:** 10.3390/plants15071126

**Published:** 2026-04-07

**Authors:** Yani Zhang, Yaxing Yang, Qiaofeng Xie, Tao Chen, Ziyue Hong, Zhaoxin Hu, Shengwu Hu

**Affiliations:** 1 College of Agronomy, Northwest A&F University, Xianyang 712100, China; zhangyani813@163.com (Y.Z.); 19232450991@163.com (Y.Y.); 3127776357@nwafu.edu.cn (Q.X.); taochen@nwafu.edu.cn (T.C.); ziyuehong2024@163.com (Z.H.); 2 Department of Electrical and Computer Engineering, University of California San Diego, La Jolla, CA 92093, USA; z1hu@eng.ucsd.edu

**Keywords:** *Brassica* species, acetohydroxyacid synthase, genetic origin, haplotype effects

## Abstract

Acetohydroxy acid synthase (AHAS) are key targets for herbicide resistance breeding in *Brassica* crops, yet the evolutionary origin and functional role of *AHAS2* genes in *Brassica napus* (AACC) and *B. carinata* (BBCC) remain poorly understood. Here, we investigated the distribution, ancestry, and agronomic trait associations of *AHAS2* across 227 accessions representing six *Brassica* species. *Bra.AHAS2* was amplified in 21 of 42 *B. rapa* (AA) accessions, and *Bol.AHAS2* in 10 of 15 *B. oleracea* (CC) accessions. In *B. napus*, *BnaA.AHAS2* and *BnaC.AHAS2* were amplified in 73/131 and 30/131 accessions, respectively, with 19 accessions showing amplification of both homologs. All seven *B. carinata* accessions amplified *BcaC.AHAS2*. No *AHAS2* homologs were amplified in three *B. nigra* (BB) or 29 *B. juncea* (AABB) accessions. Phylogenetic and gene structure analyses revealed that *BnaA.AHAS2* (in *B. napus*) originated from *Bra.AHAS2* of *B. rapa*, whereas *BnaC.AHAS2* (in *B. napus*) and *BcaC.AHAS2* (in *B. carinata*) derived from *Bol.AHAS2* of *B. oleracea*. Association analysis showed the amplification of *BnaA.AHAS2* or *BnaC.AHAS2* was not associated with tribenuron-methyl resistance. However, amplification of *BnaA.AHAS2* was significantly associated with reduced plant height, branching height, silique number on the terminal raceme, seed yield per plant, and thousand-seed weight in *B. napus*. Furthermore, haplotypes of *BnaA.AHAS2* (*BnaA05g03070D*) were significantly associated with eicosenoic acid content, oleic acid content, flowering time, and cadmium translocation. Collectively, these findings resolve the diploid progenitor origins of *AHAS2* in *Brassica* allotetraploids and reveal previously unrecognized associations of *AHAS2* with agronomic and stress-related traits, offering valuable insights for molecular breeding in oilseed *Brassica* crops.

## 1. Introduction

Acetohydroxy acid synthase (AHAS, EC 2.2.1.6), also known as acetolactate synthase (ALS, EC 4.1.3.18), is the first key rate-limiting enzyme in the biosynthetic pathway of the branched-chain amino acids (leucine, isoleucine, and valine) in plants [1,2]. This enzyme is the target site for five major classes of AHAS-inhibiting herbicides: sulfonylureas, pyrimidinylthiobenzoates, triazolopyrimidines, imidazolinones, and sulfonylamino-carbonyltriazolinones [3,4,5].

The genus *Brassica* includes many important oilseed and vegetable crops. It comprises three diploid species: *Brassica rapa* (AA, *n* = 10), *B. oleracea* (CC, *n* = 9), and *B. nigra* (BB, *n* = 8); and three amphidiploid (tetraploid) species: *B. napus* (AACC, *n* = 19), *B. juncea* (AABB, *n* = 18), and *B. carinata* (BBCC, *n* = 17). These tetraploid species were formed through natural interspecific hybridization followed by spontaneous chromosome doubling among the three diploid species, a relationship known as the “Triangle of U” [6,7].

Previous studies have reported that *B. napus* possesses three functional *AHAS* genes: *BnaAHAS1*, *BnaAHAS2*, and *BnaAHAS3* [8,9,10]. While *BnaAHAS1* and *BnaAHAS3* encode AHAS enzymes essential for plant growth and development, and specific mutations in these genes confer herbicide resistance [5,11,12,13,14,15,16], the functional significance of *BnaAHAS2* remains largely unexplored. Notably, despite extensive research on *AHAS*-mediated herbicide resistance in *Brassica*, no study has investigated whether *BnaAHAS2* contributes to resistance traits or agronomic performance.

Further, the evolutionary origin of the *AHAS2* in *Brassica* amphidiploid species remains controversial. Although specific polymerase chain reaction (PCR) methods have successfully amplified *AHAS* homologs in various *Brassica* species [17,18], conflicting reports exist regarding its progenitor origin. One study suggested that *BnaAHAS2* originated from the A genome of *B. rapa* [8,17], while others proposed that the *AHAS2* genes in both *B. napus* and *B. carinata* derived from the C genome of *B. oleracea* [18,19]. This ambiguity highlights the need for a comprehensive phylogenetic analysis across multiple *Brassica* species to resolve the evolutionary history of *AHAS2*.

In this study, we address these knowledge gaps by combining bioinformatic analysis with specific PCR amplification to investigate 227 accessions representing six *Brassica* species (*B. rapa*, *B. oleracea*, *B. nigra*, *B. napus*, *B. carinata*, and *B. juncea*). Our objectives were to: 1) determine the presence and distribution of the *AHAS2* gene among these accessions; 2) clone and sequence *AHAS2* from 10 representative accessions spanning different *Brassica* species to elucidate its phylogenetic origins; and 3) assess, through candidate gene association analysis in a panel of 131 *B. napus* accessions, whether *AHAS2* is associated with resistance to tribenuron-methyl herbicide and key agronomic traits. By systematically characterizing *AHAS2* across the U-triangle species and evaluating its phenotypic associations in *B. napus*, this study provides novel insights into the evolution and potential agronomic relevance of this previously understudied *AHAS* paralog.

## 2. Results

### 2.1. Bioinformatics Characterization of the AHAS2 Genes in Brassica Species

Using *the Arabidopsis thaliana AHAS* gene (*At3g48560*) as a reference, BLAST searches (version+2.16.0) were performed against the BRAD 3.0 (Brassica Database, http://brassicadb.cn, accessed on 28 March 2024), NCBI (National Center for Biotechnology Information, https://www.ncbi.nlm.nih.gov, accessed on 28 March 2024), and BnIR 1.0 (*Brassica napus* Multi-Omics Information Resource, https://yanglab.hzau.edu.cn/BnIR, accessed on 28 March 2024) databases to identify *AHAS2* orthologs in six *Brassica* species. In *B. oleracea*, among two sequenced accessions (*B. oleracea* JZS V2.0 and HDEM V1.0), *Bol.AHAS2* (*Bol006943*) was identified exclusively in HDEM V1.0, localized to chromosome C04 (coordinates: 2,689,242–2,691,155). In *B. rapa,* among three accessions (*B. rapa* Z1 (yellow sarson), Pakchoi V1.0, and Chiifu V4), *Bra.AHAS2* was detected in two: Z1 (yellow sarson) (BraA05t19319Z; A05: 7,666,805–7,668,718), Pakchoi V1.0 (*BraA05g00277P*; A05: 1,524,411–1,526,324). In *B. napus*, among nine sequenced cultivars, *BnaA.AHAS2* was identified in three: Darmor (*BnaA05g03070D*; A05: 1,758,226–1,760,139), Westar (*BnaA05T0024300WE*; A05: 1,394,387–1,396,300), and Quinta (*BnaA05T0031000QU*; A05: 1,824,188–1,826,180). Collectively, *BnaA.AHAS2* (*B. napus*), *Bra.AHAS2* (*B. rapa*), and *Bol.AHAS2* (*B. oleracea*) were mapped to chromosomes A05, A05, and C04, respectively.

The coding sequences of *Bna.AHAS2*, *Bol.AHAS2*, and *Bra.AHAS2* were translated to their corresponding protein sequences and analyzed using the Expasy Compute pI/Mw tool 3.0 to determine their isoelectric points (pI) and molecular weight (Mw). All three proteins (Bna.AHAS2, Bol.AHAS2, and Bra.AHAS2) shared an identical length of 637 amino acid residues. Bna.AHAS2 and Bra.AHAS2 exhibited identical molecular weight (Mw: 69,970.38 Da) and isoelectric points (pI: 5.91). Bol.AHAS2 had a Mw of 69,9942.21 Da and a pI of 5.79. Pairwise sequence comparison revealed high similarity among the three proteins, with only 21 amino acid differences between Bol.AHAS2 and the identical BnaAHAS2/BraAHAS2 pair. Subcellular localization prediction using Cell-PLoc 2.0 indicated that all three AHAS2 proteins are targeted to chloroplast.

To elucidate the evolutionary relationships among *Bna.AHAS2*, *Bol.AHAS2*, and *Bra.AHAS2*, gene structure and conserved domain analyses were performed using TBtools 2.136, with *AtAHAS* (*Arabidopsis thaliana AHAS*) as the reference. The results revealed that all three *AHAS2* genes are intronless (Figure S1c). Domain analysis demonstrated that Bna.AHAS2, Bra.AHAS2, and AtAHAS share the conserved domain PLN02470, while Bol.AHAS2 contains domain PRK08155. Both domains belong to the acetolactate synthase large subunit family (Figure S1b). Furthermore, conserved motif analysis of the three AHAS2 proteins was conducted using the MEME suite, which identified ten conserved motifs (designated motifs 1–10) (Figure S1a).

To investigate the regulatory potential of *Bna.AHAS2*, *Bol.AHAS2,* and *Bra.AHAS2*, the 1.5 kb upstream sequences of these genes were extracted and analyzed for cis-acting elements using PlantCare. The predicated elements fell into four major categories: light-responsive, hormone-related, growth/development-associated, and stress-responsive. *Bol.AHAS2* contains elements from all four categories. In contrast, *Bna.AHAS2* and *Bra.AHAS2* lacked stress-responsive elements but harbored representatives from the other three categories. The cis-element profiles of *Bna.AHAS2* and *Bra.AHAS2* were nearly identical, differing only in the presence of a light-responsive AE-box (Table S2; Figure S2). Overall, the upstream regulatory architectures of these genes showed high similarity, with *Bna.AHAS2* and *Bra.AHAS2* exhibiting near-identical profiles.

### 2.2. Analysis of AHAS2 in Tested Brassica Species

**Figure 1:**
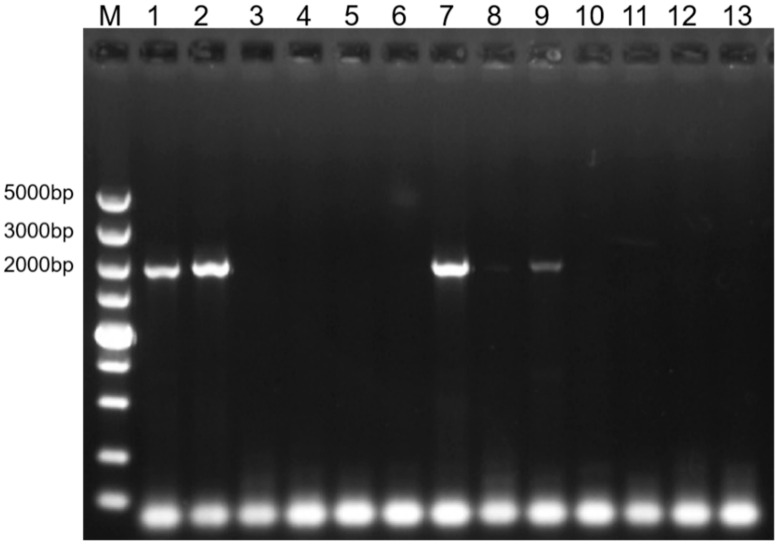
PCR amplification of *Bra.AHAS2* and its orthologs across *Brassica* species. M, DS5000 marker; 1, 0B82 (*B. rapa*, AA); 2, 0B88 (*B. rapa*, AA); 3, Qiutian-2 (*B. oleracea*, CC); 4, Yuyiganlan F_1_ (*B. oleracea*, CC); 5, 2E71 (*B. carinata*, BBCC); 6, 2E75 (*B. carinata*, BBCC); 7, 1C25 (*B. napus*, AACC); 8, 2E37 (*B. napus*, AACC); 9, 1C233 (*B. napus*, AACC); 10, 1B03 (*B. juncea*, AABB); 11, 1B16 (*B. juncea*, AABB); 12, 2E80 (*B. nigra*, BB);13, 2E81 (*B. nigra*, BB).

Two accessions each of *B. rapa* (0B82, 0B88), *B. oleracea* (Qiutian-2, Yuyi ganlan F_1_), *B. carinata* (2E71, 2E75), *B. juncea* (1B03, 1B16), and *B. nigra* (2E80, 2E81), along with three accessions of *B. napus* (1C25, 2E37, and 1C233), were subjected to PCR amplification using two primer pairs: BnALS2-F1/BnALS2-R1 and BolAHAS2-F1/BolAHAS2-R2 (Table 1). The primer pair BnALS2-F1/BnALS2-R1 amplified target products in both *B. rapa* accessions and in two of the three *B. napus* accessions (1C25 and 1C233) (Figure 1). No amplification products were observed in *B. juncea* or *B. nigra* accessions (Figure 1). These results indicate that BnALS2-F1/BnALS2-R1 is an A-genome-specific primer pair.

**Figure 2:**
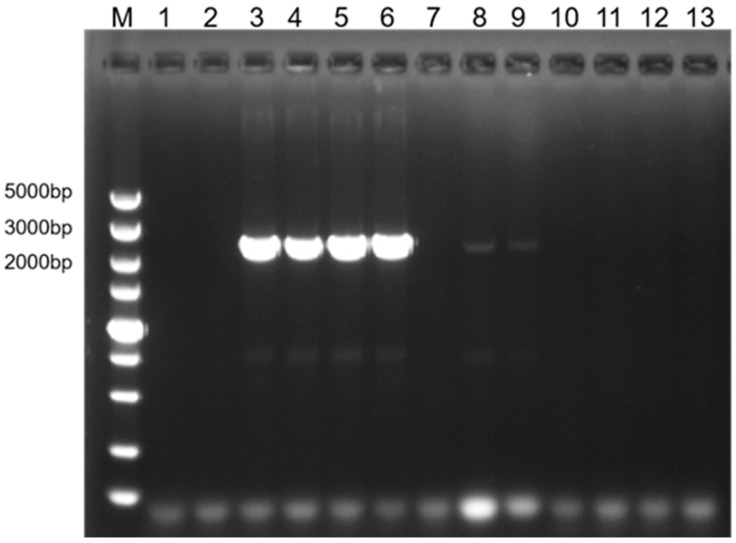
PCR amplification of *Bol.AHAS2* and its orthologs in *Brassica* species. M, DS5000 marker; 1, 0B82 (*B. rapa*, AA); 2, 0B88 (*B. rapa*, AA); 3, Qiutian-2 (*B. oleracea*, CC); 4, Yuyiganlan F_1_ (*B. oleracea*, CC); 5, 2E71 (*B. carinata*, BBCC); 6, 2E75 (*B. carinata*, BBCC); 7, 1C25 (*B. napus*, AACC); 8, 2E37 (*B. napus*, AACC); 9, 1C233 (*B. napus*, AACC); 10, 1B03 (*B. juncea*, AABB); 11, 1B16 (*B. juncea*, AABB); 12, 2E80 (*B. nigra*, BB); 13, 2E81 (*B. nigra*, BB).

The primer pair BolAHAS2-F1/BolAHAS2-R2 amplified target fragments in two accessions of *B. oleracea* and two accessions of *B. carinata*, but showed weak amplification in two *B. napus* accessions (2E37 and 1C233) (Figure 2). No amplification products were detected in *B. juncea* or *B. nigra* accessions (Figure 2). These results indicate that BolAHAS2-F1/BolAHAS2-R2 is a C-genome-specific primer pair.

Utilizing the two specific primer pairs (BnALS2-F1/BnALS2-R1 and BolAHAS2-F1/BolAHAS2-R2; Table 1), we performed *AHAS2* gene amplification in a panel of 227 *Brassica* accessions, comprising 131 *B. napus*, 42 *B. rapa*, 15 *B. oleracea*, 7 *B. carinata*, 29 *B. juncea*, and 3 *B. nigra*. Among the 15 *B. oleracea* accessions, 10 (66.67%) amplified the expected *Bol.AHAS2* product (Figure S3). For *B. rapa*, 21 of 42 (50.00%) accessions tested positive for *Bra.AHAS2* (Figure S4). All seven *B. carinata* accessions successfully amplified *BcaC.AHAS2* (Figure S5). In the 131 *B. napus* accessions, both homoeologs (*BnaA.AHAS2* and *BnaC.AHAS2*) were detected: 73 accessions (55.73%) produced the *BnaA.AHAS2* fragment (Figure S6), 30 (22.90%) the *BnaC.AHAS2* fragment (Figure S7), and 19 (14.50%) amplified both copies (Figures S6 and S7). In contrast, no *AHAS2* amplification product was obtained from any of the 29 *B. juncea* or 3 *B. nigra* accessions. A complete summary of the amplification results is provided in Table 2.

### 2.3. Cloning and Sequence Analysis of AHAS2

Two accessions each of *B. rapa* (0B82, 0B88) and *B. napus* (6C21, 6C242) were selected to amplify *Bra.AHAS2* and its A-genome orthologs using the primer pair BnALS2-F1/BnALS2-R1. The amplified target bands were cloned and sequenced. Sequencing revealed that the amplified products was 1984 bp in length in both *B. napus* accessions and in one *B. rapa* accession (0B88), with a coding sequence (CDS) of 1914 bp. However, the product from *B. rapa* accession 0B82 exhibited a 9 bp deletion at positions 699–707 bp, resulting in a CDS of 1905 bp, and the loss of three amino acids (Asp-Val-Gln) in the encoded protein. In addition to this InDel, two SNPs were identified among the four accessions: a synonymous substitution at position 1107 bp (G/C) and a missense mutation at position 1625 bp (A/G). The missense mutation resulted in an amino acid change from histidine to arginine (His→Arg) (Table S3; Supplementary File S1). Based on these sequence variations, *Bra.AHAS2* and its orthologs in *B. napus* accessions were classified into four haplotypes, including the reference haplotype_0 (Z11525.1) (Table S3).

For amplification of *Bol.AHAS2* and its C-genome orthologs, two accessions each of *B. oleracea* (Qiutian-2, Xiaguang), *B. napus* (2E35, 2E36), and *B. carinata* (2E71, 2E72) were subjected to PCR analysis using the primer pair BolAHAS2-F1/BolAHAS2-R2. Following cloning and sequencing, the amplified products were found to be 2251 bp in length across six accessions (Table S4; Supplementary File S2), with a CDS of 1914 bp. Sequence analysis identified two SNPs: a synonymous substitution at position 558 bp (T/C) and a missense mutation at position 1733 bp (T/C), the latter resulting in an amino acid change from leucine to serine (Leu→Ser) (Table S4; Supplementary File S2). These sequence variations allowed the classification of *Bol.AHAS2* and its orthologs in *B. napus* and *B. carinata* into three haplotypes, including the reference haplotype_0 (*Bol006943*) (Table S4). The sequences of the 10 cloned *AHAS2* genes have been deposited in the NCBI database under accessions numbers PX024424-PX024433.

### 2.4. Phylogenetic Analysis of AHAS2

A phylogenetic tree was constructed using the protein sequences encoded by the 10 cloned *AHAS2* genes from this study and 57 reported *AHAS1*, *AHAS2*, and *AHAS3* genes, with *Arabidopsis thaliana* AHAS (encoded by *At3g48560*) as the outgroup. The resulting tree revealed four distinct groups: Group I comprised 17 AHAS1 sequences; Group II contained 25 AHAS2 sequences; Group III included 25 AHAS3 sequences; and Group IV consisted solely of the outgroup AtAHAS sequence. AHAS1 and AHAS3 exhibited the closest genetic distance, forming a sister group. This AHAS1/AHAS3 group then grouped with the AHAS2 branches, while the AtAHAS outgroup represented the deepest divergence.

Group II (AHAS2) further resolved into two subgroups: one containing AHAS2 sequences derived from the A genome of *Brassica* species, and the other containing AHAS2 sequences derived from the C genome (Figure S8). The A-genome subgroup included eight Bra.AHAS2 (from *B. rapa*) and seven BnaA.AHAS2 (from *B. napus*), confirming that *BnaA.AHAS2* in *B. napus* originated from *Bra.AHAS2* in *B. rapa*. The C-genome subgroup contained four Bol.AHAS2 (from *B. oleracea*), two BnaC.AHAS2 (from *B. napus*), and four BcaC.AHAS2 (from *B. carinata*), indicating that both *BnaC.AHAS2* in *B. napus* and *BcaC.AHAS2* in *B. carinata* originated from *Bol.AHAS2* in *B. oleracea*.

### 2.5. Relationship Between the B. napus AHAS2 Genes and Herbicide Resistance

The preceding findings establish that *B. napus* possesses two *AHAS2* homoeologs, namely *BnaA.AHAS2* and *BnaC.AHAS2*. To explore their potential involvement in herbicide resistance, we evaluated the response of 131 *B. napus* accessions to tribenuron-methyl (Table S5). Among these accessions, 73 amplified *BnaA.AHAS2*, while 58 did not. Following treatment with tribenuron-methyl at a rate of 0.6 g a.i. ha^−1^, a comparison of the two groups across five resistance indices—phytotoxicity index, inhibition ratio of leave number, inhibition ratio of leaf angle, death rate, and average membership function—revealed no significant differences (Table 3). These results suggest that amplification of *BnaA.AHAS2* is not associated with tribenuron-methyl resistance in *B. napus* under the conditions tested.

Similarly, 30 accessions amplified *BnaC.AHAS2*, while 101 did not. After herbicide treatment, no significant differences were observed between these two groups across the same set of resistance indices (Table 4). These findings suggest that the amplification of *BnaC.AHAS2* is also not associated with tribenuron-methyl resistance in *B. napus* under the conditions evaluated.

### 2.6. Relationship Between B. napus AHAS2 Genes and Major Agronomic Traits

Analysis of the relationship between *AHAS2* genes and major agronomic traits (Table S6) indicated that *B. napus* accessions with amplification of *BnaA.AHAS2* showed significant decreases (*p* < 0.01) in plant height, branching height, number of siliques on the terminal raceme, seed yield per plant, thousand-seed weight compared with those without amplification of this gene. However, no significant differences were observed between the two groups for the remaining four traits analyzed (Table 5). Regarding *BnaC.AHAS2*, amplification of this gene was not associated with significant differences in any of the nine major agronomic traits examined (Table 6).

### 2.7. Haplotype Analysis of the AHAS2 Gene BnaA05g03070D and Its Association with Traits in B. napus

Haplotype analysis of *BnaA05g03070D* using the BnIR database identified four distinct haplotypes: hap_0, hap_1, hap_2, and hap_3. Among these, hap_0 was the most prevalent, with an allele frequency of 0.818 (*n* = 1770), followed by hap_1 (0.100, *n* = 216), hap_2 (0.053, *n* = 115), and hap_3 (0.022, *n* = 48) (Figure S9a). These haplotypes were found to be significantly associated with several agronomic and quality-related traits (*p* < 0.05), including eicosenoic acid content (Figure S9c) [21], oleic acid content (Figure S9d) [21], flowering time (Figure S9e) [22], and cadmium translocation factor (Figure S9f) [23].

Furthermore, transcriptome-phenotype association analysis using the BnIR database revealed a strong positive correlation between *AHAS2* (*BnaA05g03070D*) expression and flowering time (R = 0.551, *p* = 0.00154; Figure S9b) [22].

## 3. Discussion

Herbicide-resistant crops, when paired with their corresponding herbicides, provide a cost-effective strategy for weed management. AHAS is a key target enzyme for the development of such crops, with inhibitors spanning five major herbicide classes: sulfonylureas, pyrimidinylthiobenzoates, triazolopyrimidines, imidazolinones, and sulfonylamino-carbonyltriazolinones [4,5]. Previous studies have shown that *B. napus* possesses three functional *AHAS* genes—*BnaAHAS1*, *BnaAHAS2*, and *BnaAHAS3*—and mutations at specific sites in *BnaAHAS1* and *BnaAHAS3* confer herbicide resistance [4,5,10,11,12,13,14,15,16,19]. However, the role of *BnaAHAS2* and its origin among *Brassica* species remain ambiguous and debated [8,17,18,19]. In this study, we identified *AHAS2* orthologs in *B. oleracea* (*Bol.AHAS2*), *B. rapa* (*Bra.AHAS2*), and *B. napus* (*Bna.AHAS2*) across three databases (BRAD, NCBI, and BnIR). Using A/C-genome-specific primers targeting the *AHAS2* gene, we assessed its distribution in a panel of 227 *Brassica* accessions, comprising 131 *B. napus*, 42 *B. rapa*, 15 *B. oleracea*, 7 *B. carinata*, 29 *B. juncea*, and 3 *B. nigra* (Figures S3–S7). Successful amplification was observed in *B. oleracea* (*Bol.AHAS2*), *B. rapa* (*Bra.AHAS2*), *B. napus* (*BnaA.AHAS2* and *BnaC.AHAS2*), and *B. carinata* (*BcaC.AHAS2*), but not in *B. nigra* or *B. juncea* (Table 2; Figures S3–S7). Sequencing of cloned *AHAS2* genes from representative accessions of *B. napus*, *B. rapa*, *B. oleracea*, and *B. carinata* revealed that *Bra.AHAS2* orthologs contain a 9-bp InDel and two SNPs, whereas *BolAHAS2* orthologs exhibit two SNPs (Tables S3 and S4). Phylogenetic analysis confirmed that *BnaA.AHAS2* originated from *Bra.AHAS2* (*B. rapa*), and that both *BnaC.AHAS2* (*B. napus*) and *BcaC.AHAS2* (*B. carinata*) are derived from *Bol.AHAS2* (*B. oleracea*) (Figure S8). Association analysis indicated that amplification of *BnaA.AHAS2* or *BnaC.AHAS2* in *B. napus* was not associated with seedling resistance to tribenuron-methyl herbicide (Table 3 and Table 4). By contrast, using the BnIR database, different haplotypes of *BnaA05g03070D* were significantly associated with eicosenoic acid content, oleic acid content, flowering time, and the cadmium translocation factor (Figure S9).

The evolutionary origin of *AHAS2* in *Brassica* species has remained controversial. Five *AHAS* genes (*BnaAHAS1*-*BnaAHAS5*) have been identified in *B. napus*, and it was proposed that *BnaAHAS2*, *BnaAHAS3*, and *BnaAHAS4* originated from the A genome (*B. rapa*), whereas *BnaAHAS1* and *BnaAHAS5* were derived from the C genome (*B. oleracea*) [8]. An A-genome origin for *BnAHAS2* received subsequent support [17]. In contrast, other studies suggested a C-genome origin for *BnAHAS2* [18,19]. In the present study, we performed PCR analysis using A/C-genome-specific primers targeting *AHAS2* to examine its distribution across a diverse set of *Brassica* accessions. Among the 131 *B. napus* accessions, *BnaA.AHAS2* was successfully amplified in 73 accessions, *BnaC.AHAS2* in 30, and both homoeologs in 19. For *B. rapa*, 21 of 42 accessions yielded the *Bra.AHAS2* product, while 10 of the 15 *B. oleracea* accessions produced the *Bol.AHAS2* amplicon. All seven *B. carinata* accessions tested positive for *BcaC.AHAS2* (Table 2). Phylogenetic analysis of the protein sequences encoded by the ten *AHAS2* genes cloned in this study, together with 58 previously reported AHAS sequences, resolved four distinct groups: Group I (AHAS1), Group II (AHAS2), Group III (AHAS3), and Group IV (AtAHAS). Notably, Group II (AHAS2) further subdivided into A-genome and C-genome subgroups. Combined with gene structure analysis, these findings demonstrate that there are two *AHAS2* homoeologs (*BnaA.AHAS2* and *BnaC.AHAS2*) in *B.napus*; *BnaA.AHAS2* in *B. napus* originated from *Bra.AHAS2* in *B. rapa*; and that both *BnaC.AHAS2* in *B. napus* and *BcaC.AHAS2* in *B. carinata* originated from *Bol.AHAS2* in *B. oleracea*.

Although no *AHAS2* homologous sequences were detected in the *B. carinata* genomic database [24], the present study successfully amplified the *BcaC.AHAS2* gene from seven *B. carinata* accessions using primers specific to *Bol.AHAS2* from *B. oleracea*. This discrepancy may be attributed to several factors. First, insufficient sequencing coverage might have failed to capture the *BcaC.AHAS2* sequence [24]. Alternatively, *BcaC.AHAS2* may be absent or mutated in some *B. carinata* accessions but present in others; thus, the specific accession(s) used for genome sequencing might have lacked this gene [24]. No *AHAS2* homolog was amplified in any of the 29 *B. juncea* or 3 *B. nigra* accessions examined. Among the 42 *B. rapa* accessions, 21(50%) amplified the *Bra.AHAS2* gene (Table 2). Given that *B. juncea* (AABB genome) is an allotetraploid derived from hybridization between *B. nigra* (BB genome donor) and *B. rapa* (AA genome donor), and considering evidence that *B. juncea* specifically originated from *B. rapa* ssp. *tricolaris* [25], the absence of *AHAS2* amplification in the tested *B. juncea* accessions likely reflects the loss or mutation of this gene in its *B. rapa* ssp. *tricolaris* progenitor.

Previous studies have established that *B. napus* possesses three functional *AHAS* genes: *BnaAHAS1*, *BnaAHAS2*, and *BnaAHAS3* [8,9]. Specific mutations in *BnaAHAS1* and *BnaAHAS3* are known to confer herbicide resistance. The reported mutants and their mutation sites are as follows, PM1 and M9 carry an Asp substitution for Ser at position 653 of BnAHAS1 (BnAHAS1^S653A^); PM2 and M342 carry BnAHAS3^T574L^; PN19 carries BnAHAS1^T574L^; M45 carries BnAHAS3^P197L^; M196 carries BnAHAS1^P197L^; K5 carries BnAHAS1^P197S^; and K4 carries BnAHAS3^P197S^ [4,5,10,11,12,13,14,19,26,27,28,29]. However, the functional role of *BnaAHAS2* has remained unclear [8,18]. In the present study, amplification of *BnaA.AHAS2* or *BnaC.AHAS2* showed no association with tribenuron-methyl resistance in seedlings (Table 3 and Table 4). Given that AHAS-mediated herbicide resistance typically arises from specific gain-of-function mutations, the negative result is consistent with expectations.

In the present study, amplification of *BnaA.AHAS2* was significantly associated with several agronomic traits, including reduced plant height, branching height, number of siliques on the terminal raceme, seed yield per plant, and thousand-seed weight (Table 5). Additionally, using the BnIR database, haplotypes of *BnaA05g03070D* were found to be significantly associated with eicosenoic acid content, oleic acid content, flowering time, and cadmium translocation factor (Figure S9). A previous study indicated that *AHAS2* is structurally distinct and exhibits different expression patterns from all other plant AHAS genes [9]. These findings suggest that *BnaA.AHAS2* may play a distinct physiological role unrelated to herbicide sensitivity. AHAS enzymes are involved in the biosynthesis of branched-chain amino acids (valine, leucine, and isoleucine), which are critical for plant growth and development [1]. Therefore, natural variation in *AHAS2* expression or sequence could influence amino acid metabolism, exerting pleiotropic effects on plant architecture, yield components, and seed composition. Alternatively, *AHAS2* may be linked to other regulatory networks or genes that affect these traits, either directly or through linkage disequilibrium.

It should be noted, however, that these associations were detected using simple *t*-tests in a diversity panel, which does not account for population structure or relatedness. In future studies, we plan to genotype the *B. napus* accessions using genome-wide molecular markers to enable robust control for population structure and to validate these findings.

In summary, this study elucidates the evolutionary origin and distribution of the *AHAS2* gene across *Brassica* species and provides initial insights into the potential roles of *BnaA.AHAS2* and *BnaC.AHAS2* in *B. napus*. Future investigations employing transgenic approaches (e.g., overexpression or knockout) will be valuable for further characterizing the biological functions of both genes, particularly *BnaA.AHAS2*.

## 4. Materials and Methods

### 4.1. Plant Materials, Herbicide Resistance, and Agronomic Traits Assessment

A total of 227 *Brassica* accessions were used in this study, including 131 *B. napus*, 42 *B. rapa*, 15 *B. oleracea*, 7 *B. carinata,* 29 *B. juncea*, and 3 *B. nigra* (Table S1). All plant materials were provided by College of Agronomy, Northwest A&F University.

The 131 *B. napus* accessions were evaluated for herbicide resistance and agronomic traits during the 2023–2024 grown season at the experimental field of Caoxinzhuang Farm, Northwest A&F University, Yangling, Shaanxi, China (34°16′ N, 108°4′ E, elevation 530 m). A randomized complete block design with four replications was used. Sowing was conducted on 30 September 2023, and harvesting on 19 May 2024. Each plot comprised three 2-m-long rows with a row spacing of 0.40 m and an intra-row plant spacing of 0.15 m. All the trials were managed following standard agricultural practices.

Tribenuron-methyl resistance was evaluated at the seedling stage in two replicates following the protocol described previously [30]. Briefly, at the 3–5 leaf stage, plants were treated with tribenuron-methyl at a rate of 0.6 g a.i. ha^−1^, 300 mL.ha^−1^. Leaf damage severity was assessed three days after treatment. Nine days after treatment, leaf number and leaf–ground angle were measured. In the following spring, after regrowth, the plant mortality rate was recorded. The evaluated parameters included the phytotoxicity index, inhibition ratio of leaf number, inhibition ratio of leaf angle, death rate, and average membership function value. The phytotoxicity index was calculated as follows: phytotoxicity index = ∑ representative value of a grade × number of plants in that grade)/(total number of plants × number of grades). The different levels of injury were classified into 7 grades, with 0 represents no injury, and 6 representing full damage. The membership function was calculated using the formula: S(X) = (X − Xmin)/(Xmax − Xmin), where X represents the measured value of the sample for a given trait, and Xmin and Xmax are the minimum and maximum values of that trait among all samples, respectively.

The remaining two replicates were used to evaluate key agronomic traits at maturity. For these, nine agronomic traits were measured on 10 plants sampled from the middle row of each plot: plant height, height of the first primary branch, number of siliques on the main inflorescence, siliques per plant, seeds per silique, number of effective branches, length of the main inflorescence, thousand-seed weight, and seed yield per plant. Associations between *AHAS2* genotypes and herbicide resistance as well as agronomic traits, were analyzed using Student’s *t*-test in SPSS 24.0, with groups defined based on the presence or absence of the target gene amplification.

The remaining *Brassica* accessions (*B. rapa*, *B. oleracea*, *B. juncea*, *B. carinata*, and *B. nigra*) were planted in a single replication under the same field design and management conditions. Fresh leaves were collected from each accession at the five-leaf stage. For each accession, leaves from 15 randomly selected plants were pooled and stored at −80 °C for subsequent analysis.

### 4.2. Bioinformatics Analysis of the AHAS2 Gene

DNA sequence information for the *Arabidopsis thaliana AHAS* gene (*AtAHAS*, *At3g48560*) was obtained from the TAIR database (http://www.arabidopsis.org/, accessed on 28 March 2024). The *AtAHAS* sequence was used as a reference to perform BLAST searches against three databases—BRAD 3.0 (Brassica Database, http://brassicadb.cn, accessed on 28 March 2024), NCBI (National Center for Biotechnology Information, https://www.ncbi.nlm.nih.gov, accessed on 28 March 2024), and BnIR 1.0 (*Brassica napus* Multi-Omics Information Resource, https://yanglab.hzau.edu.cn/BnIR, accessed on 28 March 2024)—to identify *AHAS2* homologous genes in six *Brassica* species: *B. oleracea*, *B. rapa*, *B. napus*, *B. carinata*, *B. juncea*, and *B. nigra*. Chromosomal locations of candidate *AHAS2* genes were generated by analyzing genome annotation files of *Brassica* species. Gene distribution maps were plotted using MapChart software 1.0 (accessed on 1 April 2024). Coding sequences (CDS) of candidate *AHAS2* genes were extracted and translated into protein sequences. Isoelectric point (pI), amino acid length, and molecular weight (Mw) were predicted using ExPASy Compute pI/Mw 3.0 (https://web.expasy.org/compute_pi/, accessed on 3 April 2024). Protein subcellular localization was predicted via Cell-PLoc 2.0 (http://www.csbio.sjtu.edu.cn/bioinf/Cell-PLoc-2/, accessed on 3 April 2024). Gene structures (intron/exon patterns) and conserved domains were analyzed with TBtools 2.136. Conserved motifs were identified via the MEME Suite 4.0 (https://meme-suite.org/meme/tools/meme, accessed on 6 April 2024) and the results were visualized in TBtools, with *AtAHAS* serving as a reference for comparative analysis. A 1.5 kb upstream sequence of each candidate *AHAS2* gene was extracted to predict *cis*-acting regulatory elements using PlantCARE 1.0 (http://bioinformatics.psb.ugent.be/webtools/plantcare/html/, accessed on 10 April 2024).

### 4.3. Cloning, Sequencing, and Sequence Alignment of the AHAS2 Gene

Total genomic DNA was extracted from 15 seedlings at five-leaf stage for each accessions using the CTAB (Cetyltrimethylammonium Bromide) method [31]. Two pairs of specific primers were designed to amplify the *AHAS2* gene from the A and C genomes of *Brassica* species, respectively (Table 1). Primers BnALS2-F1/BnALS2-R1 (targeting the A-genome *AHAS2*) were adopted from previous study [20]. Primers BolAHAS2-F1/BolAHAS2-R2 (targeting the C-genome *AHAS2*) were designed using Primer 5.0 software, based on UTR sequence alignment between the *B. oleracea* gene *Bol006943* and the *B. rapa* gene *BraA05t19319Z*. All primers were synthesized by Tsingke Biotechnology Co., Ltd. (Beijing, China).

The PCR mixture consisted of 10 μL total volume, containing 1.5 μL template DNA (150 ng μL^−1^), 1 μL each of forward and reverse primers (10 μmol L^−1^), 5 μL 2× Rapid Taq Master Mix (Vazyme, Nanjing, China), and 1.5 μL ddH_2_O. The PCR amplification was performed in a C1000 thermal cycler (Bio-Rad Co. Ltd., Hercules, CA, USA) under the following conditions: initial denaturation at 95 °C for 3 min; 33 cycles of denaturation at 95 °C for 27 s, annealing at 56 °C (for primer pair BnALS2-F1/BnALS2-R1) or 60 °C (for primer pair BolAHAS2-F1/BolAHAS2-R2) for 27 s, and extension at 72 °C for 40 s; and a final extension at 72 °C for 5 min. PCR products were separated by 1.0% agarose gel electrophoresis. The expected bands of PCR products were excised and purified using a DNA Purification kit (Tiangen, Beijing, China), and then cloned into the pMD19-T vector (Takara, Kyoto, Japan) following the manufacturer’s instructions. Plasmid DNA was isolated and sequenced by Sangon Corporation. Sequence data was analyzed using DNAMAN software Version 3.0 (Lynnon BioSoft, Cambridge, MA, USA). For each sample, five clones were randomly selected for sequencing.

### 4.4. Evolutionary Analysis of AHAS2 in Brassica Species

Nucleotide sequences of 10 cloned *AHAS2* genes were translated into protein sequences using Expasy Translate 3.0 (https://web.expasy.org/translate/, accessed on 22 October 2024). A total of 68 AHAS protein sequences were analyzed, including the 10 translated AHAS2 sequences obtained in this study and 57 of AHAS1, AHAS2, and AHAS3 sequences retrieved from NCBI and BRAD 3.0 databases. *Arabidopsis* AHAS was used as the reference. Phylogenetic analysis was performed using MEGA11. Multiple sequence alignment was conducted using “Align”, and a phylogenetic tree was subsequently constructed using the “Phylogenetic Analysis” tool. The Neighbor-Joining algorithm was employed to construct the tree, and the resulting phylogenetic tree was exported in Newick format. Visualization was performed using TBtools 2.136 (accessed on 25 October 2024).

### 4.5. Haplotype Analysis of the AHAS2 Gene BnaA05g03070D in B. napus

Based on BnIR dateset, the Single-Site Variation module was used to analyze the haplotypes of *BnaA05g03070D* and its ± 2 kb flanking regions, and to assess their effects on associated traits. Concurrently, the Transcriptome-Phenotype Association module was employed to analyze the association between the transcriptome data of *BnaA05g03070D* and phenotypic traits [32].

## Figures and Tables

**Table 1:** Primers used in the present investigation.

Primer Name	Sequence(5′~3′)	Location	The Reference Gene in GenBank	Expected Fragment Size (bp)	Reference
BnALS2-F1	AAGCAATTTCTCGCAACACTC	−32~−11bp upstream of CDS	Z11525.1	1984	[20]
BnALS2-R1	CAGAAGAGAGCATAGAATAATCAA	+14~+38 bp downstream of CDS			
BolAHAS2-F1	GTTGGTAGGTAAGATATCATTAG	−180~−158 bp upstream of CDS	*Bol006943*(C04)	2251	
BolAHAS2-R2	CATGTTCCTACTTTTAGTCG	+138~+157 bp downstream of CDS			

Note: The sequences of Z11525.1 and *Bol006943* were downloaded from NCBI (https://www.ncbi.nlm.nih.gov/, accessed on 24 March 2024) and BRAD (http://brassicadb.cn, accessed on 24 March 2024), respectively. CDS, coding sequence of the reference gene. The first base upstream of the initiation codon (ATG) is −1 and the first base downstream of the stop codon (TGA) is +1.

**Table 2:** Summary of *AHAS2* amplification across the tested *Brassica* species.

Species	*Bol.AHAS2*/*BnaC.AHAS2*/*Bca.AHAS2*	*Bra.AHAS2*/*BnaA.AHAS2*	Total
*Brassica rapa*	0	21	42
*B. oleracea*	10	0	15
*B. nigra*	0	0	3
*B. napus* ^1^	30 (19)	73 (19)	131
*B. carinata*	7	0	7
*B. juncea*	0	0	29

Note: ^1^, number of *B. napus* accessions amplifying both *BnaC.AHAS2* and *BnaA.AHAS2* alleles (shown in parentheses).

**Table 3:** Comparison of herbicide resistance between *B. napus* accessions with and without amplification of *BnaA.AHAS2*.

*BnaA.AHAS2* Amplified/Not Amplified	Phytotoxicity Index	Inhibition Ratio of Leave Number	Inhibition Ratio of Leaf Angle	Death Rate	Average Membership Function Value
Amplified (*n* = 73)	0.65 ± 0.01 a	0.32 ± 0.01 a	0.64 ± 0.01 a	95.65 ± 0.87 a	0.66 ± 0.02 a
Not amplified (*n* = 58)	0.66 ± 0.02 a	0.31 ± 0.01 a	0.62 ± 0.01 a	95.91 ± 0.912 a	0.62 ± 0.02 a

Note: Data are expressed by mean ± SE (standard error). Values within the same column followed by the same lowercase letter are not significantly different.

**Table 4:** Comparison of herbicide resistance between *B. napus* accessions with and without amplification of *BnaC.AHAS2*.

*BnaC.AHAS2* Amplified/Not Amplified	Phytotoxicity Index	Inhibition Ratio of Leave Number	Inhibition Ratio of Leaf Angle	Death Rate	Average Membership Function Value
Amplified (*n* = 30)	0.63 ± 0.03 a	0.32 ± 0.01 a	0.61 ± 0.02 a	97.78 ± 0.63 a	0.66 ± 0.03 a
Not amplified (*n* = 101)	0.66 ± 0.15 a	0.32 ± 0.01 a	0.64 ± 0.01 a	95.17 ± 0.78 a	0.63 ± 0.02 a

Note: Data are expressed by mean ± SE (standard error). Values within the same column followed by the same lowercase letter are not significantly different.

**Table 5:** Comparison of main agronomic traits between *B. napus* accessions with and without amplification of *BnaA.AHAS2*.

*BnaC.AHAS2* Amplified/Not Amplified	PH (cm)	BH (cm)	NB (cm)	LTM (cm)	NSTR	NSP	NS	TSW (g)	SYP (g)
Amplified (*n* = 30)	161.38 ± 2.63 a	56.01 ± 2.89 a	8.54 ± 0.27 a	52.82 ± 1.08 a	63.14 ± 2.11 a	278.34 ± 19.17 a	20.04 ± 0.64 a	3.05 ± 0.14 a	18.39 ± 1.38 a
Not amplified (*n* = 101)	163.84 ± 2.18 a	56.57 ± 2.42 a	8.42 ± 0.14 a	54.40 ± 0.67 a	62.70 ± 1.42 a	279.17 ± 8.59 a	20.54 ± 0.27 a	3.21 ± 0.08 a	17.06 ± 0.55 a

Note: Plant height (PH), branching height (BH), number of primary branches per plant (NB), Length of the terminal raceme (LTM), number of siliques on the terminal raceme (NSTR), number of siliques per plant (NSP), number of seeds per silique (NS), thousand-seed weight (TSW), seed yield per plant (SYP). Data are expressed by mean ± SE (standard error). Within each column, data followed by the same lowercase letter are not significantly different (*p* ≥ 0.05).

**Table 6:** Comparison of main agronomic traits between *B. napus* accessions with and without amplification of *BnaC.AHAS2*.

*BnaA.AHAS2* Amplified/Not Amplified	PH (cm)	BH (cm)	NB (cm)	LTM (cm)	NSTR	NSP	NS	TSW (g)	SYP (g)
Amplified (*n* = 73)	159.96 ± 2.47 b	52.25 ± 2.82 B	8.60 ± 0.16 a	53.10 ± 0.76 a	60.23 ± 1.55 b	262.64 ± 9.63 b	20.77 ± 0.36 a	2.99 ± 0.10 B	17.58 ± 0.75 a
Not amplified (*n* = 58)	167.45 ± 2.49 a	61.72 ± 2.57 A	8.25 ± 0.17 a	55.22 ± 0.86 a	66.04 ± 1.79 a	299.54 ± 12.71 a	20.00 ± 0.34 a	3.40 ± 0.10 A	17.09 ± 0.74 a

Note: Plant height (PH), branching height (BH), number of primary branches per plant (NB), Length of the terminal raceme (LTM), number of siliques on the terminal raceme (NSTR), number of siliques per plant (NSP), number of seeds per silique (NS), thousand-seed weight (TSW), seed yield per plant (SYP). Data are expressed by mean ± SE (standard error). Within each column, data followed by the same lowercase letter are not significantly different (*p* ≥ 0.05), whereas those followed by different capital letters differ significantly at the *p* < 0.05 or *p* < 0.01 level, respectively.

## Data Availability

The data that support the findings of this study are available in the Supplementary Material of this article and the sequences of the 10 cloned *AHAS2* genes have been deposited in the NCBI database (https://www.ncbi.nlm.nih.gov, accessions numbers PX024424-PX024433).

## Supplementary Materials

The following supporting information can be downloaded at: https://www.mdpi.com/article/10.3390/plants15071126/s1, Figure S1. *AHAS2* gene structure and conserved domain of its encoded proteins. (a) the conserved motif, designated motifs 1–10; (b) conserved domain, PLN22470 and PRK08155 superfamily belong to the acetolactate synthase large subunit family; (c) gene structure. Figure S2. Analysis of cis-acting elements in the 1.5 kb upstream sequences of *Bra.AHAS2*, *Bna.AHAS2* and *Bol.AHAS2* genes. Figure S3. PCR amplification of *Bol.AHAS2* in *B*. *oleracea* (CC) accessions. M, DS5000 marker; the accession numbers 174~188 are shown in Table S1. Figure S4. PCR amplification of *Bra.AHAS2* in *B*. *rapa* (AA) accessions. M, DS5000 marker; the accession numbers 132~173 are shown in Table S1. Figure S5. PCR amplification of *BcaC.AHAS2* in *B*. *carinata* (BBCC) accessions. M, DS5000 marker; the accession numbers 189~195 are shown in Table S1. Figure S6. PCR amplification of *BnaA.AHAS2* in *B*. *napus* (AACC) accessions. M, DS5000 marker; the accession numbers 1~131 are shown in Table S1. Figure S7. PCR amplification of *BnaC.AHAS2* in *B*. *napus* (AACC) accessions. M, DS5000 marker; the accession numbers 1~131 are shown in Table S1. Figure S8. Phylogenetic analysis of AHAS2 in *Brassica* crops. All AHAS2 proteins are grouped into four branches, and each branch is represented by a different color. The red branch indicates that AHAS from *Arabidopsis thaliana*; the yellow branch indicates that AHAS1 from *Brassica*; the green branch indicates that AHAS2 from *Brassica*; the blue branch indicates that AHAS3 from *Brassica*. AtAHAS sequence from the TAIR database; sequences 0B82, 0B88, 6C21, 6C242, Qiutian-2, Xiaguang, 2E35, 2E36, 2E71, and 2E72 were obtained in the present study; other gene sequences from the NCBI database. Figure S9. *BnaA05g03070D* haplotype effect on associated traits and transcriptome-phenotype correlation analysis in *B*. *napus* accessions using the BnIR dataset. (a) Haplotype information of *BnaA05g03070D* and its ± 2 kb flanking region. (b) Correlation between *BnaA05g03070D* expression and flowering time. Haplotype effect of *BnaA05g03070D* on (c) flowering time, (d) oleic acid, (e) eicosenoic acid, and (f) cd translocation coefficient. Table S1. Plant materials used in the present investigation. Table S2. Upstream *cis*-acting elements of *Bra.AHAS2*, *Bna.AHAS2* and *Bol.AHAS2* genes. Table S3. Comparison of DNA and protein sequences of *Bra.AHAS2* and its orthologs across *Brassica* species. Table S4. Comparison of DNA and protein sequences of *Bol.AHAS2* and its orthologs across *Brassica* species. Table S5. Herbicide resistance of 131 *B. napus* accessions. Table S6. Main agronomic traits of 131 *B. napus* accessions. Supplementary File S1. DNA sequences of *Bra.AHAS2* and its orthologs in *Brassica* species. Supplementary File S2. DNA sequences of *Bol.AHAS2* and its orthologs in *Brassica* species.
