# Supplementary material for: Genetic Origin of AHAS2 Genes in Brassica Allotetraploids and Association of Its Orthologs with Agronomic Traits in B. napus
Source: Plants (Basel). 2026 Apr 7;15(7):1126. doi: 10.3390/plants15071126 (PMC13074368; doi:10.3390/plants15071126)
Supplement: Supplementary file 1 [file plants-15-01126-s001.zip › Supplementary file S1.pdf]

Supplementary file 1. DNA sequences of *Bra.AHAS2* and orthologs in *Brassica* species

6C21

SEQ DNAMAN1: 1987 bp;

Composition 460 A; 525 C; 501 G; 501 T; 0 OTHER

Percentage: 23% A; 26% C; 25% G; 25% T; 0%OTHER

Molecular Weight (kDa): ssDNA: 612.84 dsDNA: 1225.0

ORIGIN

1 ATTAAGCAAT TTCTCGCAAC ACTCCATTTT GCACCATGGC TTCGTTTTCG TTCTTCGGCA

61 CCATTCCGTC GTCTCCCACA AAAGCTTCCG TCTTCTCCCT GCCGGTGTCG GTAACTACGC

121 TCCCGTCCTT CCCGCGCCGC CGTGCTACTC GTGTCTCCGT TTCCGCCAAC TCGAAGAAAG

181 ACCAAGACCG CACAGCTTCA CGTCGAGAGA ATCCGAGCAC ATTCAGCTCC AAATACGCTC

241 CCAACGTGCC CCGCAGTGGC GCAGACATCC TGGTCGAAGC CCTGGAGCGT CAAGGAGTGG

301 ACGTAGTCTT CGCTTACCCA GGAGGCGCAT CAATGGAGAT CCATCAAGCC CTAACTCGCT

361 CCAACACAAT CCGAAACGTC CTTCCCCGTC ACGAACAAGG AGGTATCTTC GCCGCCGAGG

421 GTTACGCTCG TTCCTCCGGT AAACCCGGAA TCTGCATCGC CACTTCCGGT CCAGGAGCTA

481 TGAATCTCGT CAGCGGATTA GCCGACGCCC TGTTTGACAG CGTACCCCTC ATCGCAATCA

541 CAGGACAGGT CCCTCGCCGG ATGATTGGTA CCATGGCGTT CCAGGAGACA CCCGTTGTTG

601 AGGTAACGAG GACTATAACG AAACATAACT ATCTTGTTAT GGAAGTTGAT GATATACCTA

661 GGATCGTTCG AGAAGCTTTC TTTCTAGCTA CTTCGGTTAG ACCGGGACCG GTTCTTATAG

721 ACGTCCCCAA AGATGTTCAG CAACAGTTTG CGATTCCTAA CTGGGAACAG CCTATGCGCT

781 TACCTCTTTA CATGTCTACG ATGCCTAAAC CCCCCAAAGT TTCTCACTTA GAGCAGATTC

841 TTAGGTTGGT TTCGGAGTCT AAGAGGCCTG TCTTGTACGT TGGAGGTGGT TGTCTGAACT

901 CGAGTGAGGA ACTGCGCAGA TTTGTGGAAC TTACTGGCAT CCCTGTTGCT AGTACGTTCA

961 TGGGACTTGG ATCGTATCCT TGTGACGATG AAGAGTTCTC TCTGCAAATG CTAGGAATGC

1021 ATGGAACAGT GTACGCTAAT TACGCTGTCG AGTATAGCGA TCTTCTGCTT GCTTTTGGGG

1081 TTAGGTTTGA CGACCGTGTG ACCGGAAAGC TTGAGGCCTT TGCTAGCCGG GCCAAGATCG
1141 TGCATATTGA TATTGATTCT ACCGAAATCG GGAAGAACAA GACACCTCAT GTGTCGGTGT
1201 GTTGTGATGT TCAGCTAGCC TTGCAAGGGA TGAACGAGGT TCTTGAGAAC CGACGAGATG
1261 TGCTTGACTT CGGGGAATGG AGATGTGAAT TGAACGAACA GAGACTAAAG TTCCCTCTCC
1321 GCTACAAGAC GTTTGGGGAA GAGATTCCTC CACAGTACGC CATTCAACTA CTTGACGAGC
1381 TAACCGACGG GAAGGCAATT ATCACTACTG GTGTCGGGCA ACACCAGATG TGGGCCGCCC
1441 AATTCTACAG ATTCAAGAAA CCCCGCCAAT GGCTGTCTTC AGGAGGCCTA GGAGCCATGG
1501 GGTTCGGTCT TCCTGCAGCC ATGGGAGCCG CTATAGCCAA CCCGGGAGCA GTGGTTGTCG
1561 ACATTGATGG GGATGGTAGC TTCATCATGA ACATTCAAGA ACTGGCAACC ATCAGGGTTG
1621 AGAATCTTCC AGTCAAGGTT TTGCTGATTA ATAATCAGCA CCTCGGAATG GTCCTTCAGT
1681 GGGAAGACCA CTTCTACGCA GCTAACAGAG CCGATTCTTT TCTGGGAGAC CCGGCGAACC
1741 CTGAGGCGGT ATTCCCGGAT ATGCTGTTGT TCGCCGCATC GTGCGGTATA CCAGCCGCCA
1801 GGGTCACCAG AAGGGAGGAC CTCCGAGAGG CAATCCAGAC AATGCTGGAC ACACCTGGAC
1861 CATTCTTGTT GGATGTGGTC TGTCCTCACC AGGACCATGT GTTACCACTC ATCCCTAGTG
1921 GCGGCACCTT CAAGGACATT ATTGTGTAGT ACTACTTTAA TGTTTGATTA TTCTATGCTC
1981 TCTTCTG

6C242

SEQ DNAMAN2: 1987 bp;

Composition 460 A; 525 C; 501 G; 501 T; 0 OTHER

Percentage: 23% A; 26% C; 25% G; 25% T; 0%OTHER

Molecular Weight (kDa): ssDNA: 612.84 dsDNA: 1225.0

ORIGIN

1 ATTAAGCAAT TTCTCGCAAC ACTCCATTTT GCACCATGGC TTCGTTTTCG TTCTTCGGCA
61 CCATTCCGTC GTCTCCCACA AAAGCTTCCG TCTTCTCCCT GCCGGTGTCG GTAACTACGC
121 TCCCGTCCTT CCCGCGCCGC CGTGCTACTC GTGTCTCCGT TTCCGCCAAC TCGAAGAAAG

181 ACCAAGACCG CACAGCTTCA CGTCGAGAGA ATCCGAGCAC ATTCAGCTCC AAATACGCTC

241 CCAACGTGCC CCGCAGTGGC GCAGACATCC TGGTCGAAGC CCTGGAGCGT CAAGGAGTGG

301 ACGTAGTCTT CGCTTACCCA GGAGGCGCAT CAATGGAGAT CCATCAAGCC CTAACTCGCT

361 CCAACACAAT CCGAAACGTC CTTCCCCGTC ACGAACAAGG AGGTATCTTC GCCGCCGAGG

421 GTTACGCTCG TTCCTCCGGT AAACCCGGAA TCTGCATCGC CACTTCCGGT CCAGGAGCTA

481 TGAATCTCGT CAGCGGATTA GCCGACGCCC TGTTTGACAG CGTACCCCTC ATCGCAATCA

541 CAGGACAGGT CCCTCGCCGG ATGATTGGTA CCATGGCGTT CCAGGAGACA CCCGTTGTTG

601 AGGTAACGAG GACTATAACG AAACATAACT ATCTTGTTAT GGAAGTTGAT GATATACCTA

661 GGATCGTTCG AGAAGCTTTC TTTCTAGCTA CTTCGGTTAG ACCGGGACCG GTTCTTATAG

721 ACGTCCCCAA AGATGTTCAG CAACAGTTTG CGATTCCTAA CTGGGAACAG CCTATGCGCT

781 TACCTCTTTA CATGTCTACG ATGCCTAAAC CCCCCAAAGT TTCTCACTTA GAGCAGATTC

841 TTAGGTTGGT TTCGGAGTCT AAGAGGCCTG TCTTGTACGT TGGAGGTGGT TGTCTGAACT

901 CGAGTGAGGA ACTGCGCAGA TTTGTGGAAC TTACTGGCAT CCCTGTTGCT AGTACGTTCA

961 TGGGACTTGG ATCGTATCCT TGTGACGATG AAGAGTTCTC TCTGCAAATG CTAGGAATGC

1021 ATGGAACAGT GTACGCTAAT TACGCTGTCG AGTATAGCGA TCTTCTGCTT GCTTTTGGGG

1081 TTAGGTTTGA CGACCGTGTG ACCGGAAAGC TTGAGGCCTT TGCTAGCCGG GCCAAGATCG

1141 TGCATATTGA TATTGATTCT ACCGAAATCG GGAAGAACAA GACACCTCAT GTGTCGGTGT

1201 GTTGTGATGT TCAGCTAGCC TTGCAAGGGA TGAACGAGGT TCTTGAGAAC CGACGAGATG

1261 TGCTTGACTT CGGGGAATGG AGATGTGAAT TGAACGAACA GAGACTAAAG TTCCCTCTCC

1321 GCTACAAGAC GTTTGGGGAA GAGATTCCTC CACAGTACGC CATTCAACTA CTTGACGAGC

1381 TAACCGACGG GAAGGCAATT ATCACTACTG GTGTCGGGCA ACACCAGATG TGGGCCGCCC

1441 AATTCTACAG ATTCAAGAAA CCCCGCCAAT GGCTGTCTTC AGGAGGCCTA GGAGCCATGG

1501 GGTTCGGTCT TCCTGCAGCC ATGGGAGCCG CTATAGCCAA CCCGGGAGCA GTGGTTGTCG
1561 ACATTGATGG GGATGGTAGC TTCATCATGA ACATTCAAGA ACTGGCAACC ATCAGGGTTG
1621 AGAATCTTCC AGTCAAGGTT TTGCTGATTA ATAATCAGCA CCTCGGAATG GTCCTTCAGT
1681 GGGAAGACCA CTTCTACGCA GCTAACAGAG CCGATTCTTT TCTGGGAGAC CCGGCGAACC
1741 CTGAGGCGGT ATTCCCGGAT ATGCTGTTGT TCGCCGCATC GTGCGGTATA CCAGCCGCCA
1801 GGGTCACCAG AAGGGAGGAC CTCCGAGAGG CAATCCAGAC AATGCTGGAC ACACCTGGAC
1861 CATTCTTGTT GGATGTGGTC TGTCCTCACC AGGACCATGT GTTACCACTC ATCCCTAGTG
1921 GCGGCACCTT CAAGGACATT ATTGTGTAGT ACTACTTTAA TGTTTGATTA TTCTATGCTC
1981 TCTTCTG

0B88

SEQ DNAMAN3: 1987 bp;

Composition 459 A; 526 C; 501 G; 501 T; 0 OTHER

Percentage: 23% A; 26% C; 25% G; 25% T; 0%OTHER

Molecular Weight (kDa): ssDNA: 612.81 dsDNA: 1225.0

ORIGIN

1 ATTAAGCAAT TTCTCGCAAC ACTCCATTTT GCACCATGGC TTCGTTTTCG TTCTTCGGCA
61 CCATTCCGTC GTCTCCCACA AAAGCTTCCG TCTTCTCCCT GCCGGTGTCG GTAACTACGC
121 TCCCGTCCTT CCCGCGCCGC CGTGCTACTC GTGTCTCCGT TTCCGCCAAC TCGAAGAAAG
181 ACCAAGACCG CACAGCTTCA CGTCGAGAGA ATCCGAGCAC ATTCAGCTCC AAATACGCTC
241 CCAACGTGCC CCGCAGTGGC GCAGACATCC TGGTCGAAGC CCTGGAGCGT CAAGGAGTGG
301 ACGTAGTCTT CGCTTACCCA GGAGGCGCAT CAATGGAGAT CCATCAAGCC CTAACTCGCT
361 CCAACACAAT CCGAAACGTC CTTCCCCGTC ACGAACAAGG AGGTATCTTC GCCGCCGAGG
421 GTTACGCTCG TTCCTCCGGT AAACCCGGAA TCTGCATCGC CACTTCCGGT CCAGGAGCTA
481 TGAATCTCGT CAGCGGATTA GCCGACGCCC TGTTTGACAG CGTACCCCTC ATCGCAATCA
541 CAGGACAGGT CCCTCGCCGG ATGATTGGTA CCATGGCGTT CCAGGAGACA CCCGTTGTTG

601 AGGTAACGAG GACTATAACG AAACATAACT ATCTTGTTAT GGAAGTTGAT GATATACCTA

661 GGATCGTTCG AGAAGCTTTC TTTCTAGCTA CTTCGGTTAG ACCGGGACCG GTTCTTATAG

721 ACGTCCCCAA AGATGTTCAG CAACAGTTTG CGATTCCTAA CTGGGAACAG CCTATGCGCT

781 TACCTCTTTA CATGTCTACG ATGCCTAAAC CCCCCAAAGT TTCTCACTTA GAGCAGATTC

841 TTAGGTTGGT TTCGGAGTCT AAGAGGCCTG TCTTGTACGT TGGAGGTGGT TGTCTGAACT

901 CGAGTGAGGA ACTGCGCAGA TTTGTGGAAC TTACTGGCAT CCCTGTTGCT AGTACGTTCA

961 TGGGACTTGG ATCGTATCCT TGTGACGATG AAGAGTTCTC TCTGCAAATG CTAGGAATGC

1021 ATGGAACAGT GTACGCTAAT TACGCTGTCG AGTATAGCGA TCTTCTGCTT GCTTTTGGGG

1081 TTAGGTTTGA CGACCGTGTG ACCGGAAAGC TTGAGGCCTT TGCTAGCCGG GCCAAGATCG

1141 TCCATATTGA TATTGATTCT ACCGAAATCG GGAAGAACAA GACACCTCAT GTGTCGGTGT

1201 GTTGTGATGT TCAGCTAGCC TTGCAAGGGA TGAACGAGGT TCTTGAGAAC CGACGAGATG

1261 TGCTTGACTT CGGGGAATGG AGATGTGAAT TGAACGAACA GAGACTAAAG TTCCCTCTCC

1321 GCTACAAGAC GTTTGGGGAA GAGATTCCTC CACAGTACGC CATTCAACTA CTTGACGAGC

1381 TAACCGACGG GAAGGCAATT ATCACTACTG GTGTCGGGCA ACACCAGATG TGGGCCGCCC

1441 AATTCTACAG ATTCAAGAAA CCCCGCCAAT GGCTGTCTTC AGGAGGCCTA GGAGCCATGG

1501 GGTTCGGTCT TCCTGCAGCC ATGGGAGCCG CTATAGCCAA CCCGGGAGCA GTGGTTGTCG

1561 ACATTGATGG GGATGGTAGC TTCATCATGA ACATTCAAGA ACTGGCAACC ATCAGGGTTG

1621 AGAATCTTCC AGTCAAGGTT TTGCTGATTA ATAATCAGCG CCTCGGAATG GTCCTTCAGT

1681 GGGAAGACCA CTTCTACGCA GCTAACAGAG CCGATTCTTT TCTGGGAGAC CCGGCGAACC

1741 CTGAGGCGGT ATTCCCGGAT ATGCTGTTGT TCGCCGCATC GTGCGGTATA CCAGCCGCCA

1801 GGGTCACCAG AAGGGAGGAC CTCCGAGAGG CAATCCAGAC AATGCTGGAC ACACCTGGAC

1861 CATTCTTGTT GGATGTGGTC TGTCCTCACC AGGACCATGT GTTACCACTC ATCCCTAGTG

1921 GCGGCACCTT CAAGGACATT ATTGTGTAGT ACTACTTTAA TGTTTGATTA TTCTATGCTC
1981 TCTTCTG

0B87

SEQ DNAMAN4: 1978 bp;
Composition 458 A; 523 C; 499 G; 498 T; 0 OTHER
Percentage: 23% A; 26% C; 25% G; 25% T; 0%OTHER
Molecular Weight (kDa): ssDNA: 610.06 dsDNA: 1219.5
ORIGIN
1 ATTAAGCAAT TTCTCGCAAC ACTCCATTTT GCACCATGGC TTCGTTTTCG TTCTTCGGCA
61 CCATTCCGTC GTCTCCCACA AAAGCTTCCG TCTTCTCCCT GCCGGTGTCG GTAACTACGC
121 TCCCGTCCTT CCCGCGCCGC CGTGCTACTC GTGTCTCCGT TTCCGCCAAC TCGAAGAAAG
181 ACCAAGACCG CACAGCTTCA CGTCGAGAGA ATCCGAGCAC ATTCAGCTCC AAATACGCTC
241 CCAACGTGCC CCGCAGTGGC GCAGACATCC TGGTCGAAGC CCTGGAGCGT CAAGGAGTGG
301 ACGTAGTCTT CGCTTACCCA GGAGGCGCAT CAATGGAGAT CCATCAAGCC CTAACTCGCT
361 CCAACACAAT CCGAAACGTC CTTCCCCGTC ACGAACAAGG AGGTATCTTC GCCGCCGAGG
421 GTTACGCTCG TTCCTCCGGT AAACCCGGAA TCTGCATCGC CACTTCCGGT CCAGGAGCTA
481 TGAATCTCGT CAGCGGATTA GCCGACGCCC TGTTTGACAG CGTACCCCTC ATCGCAATCA
541 CAGGACAGGT CCCTCGCCGG ATGATTGGTA CCATGGCGTT CCAGGAGACA CCCGTTGTTG
601 AGGTAACGAG GACTATAACG AAACATAACT ATCTTGTTAT GGAAGTTGAT GATATACCTA
661 GGATCGTTCG AGAAGCTTTC TTTCTAGCTA CTTCGGTTAG ACCGGGACCG GTTCTTATAG
721 ACGTCCCCAA AGAACAGTTT GCGATTCCTA ACTGGGAACA GCCTATGCGC TTACCTCTTT
781 ACATGTCTAC GATGCCTAAA CCCCCCAAAG TTTCTCACTT AGAGCAGATT CTTAGGTTGG
841 TTTCGGAGTC TAAGAGGCCT GTCTTGTACG TTGGAGGTGG TTGTCTGAAC TCGAGTGAGG
901 AACTGCGCAG ATTTGTGGAA CTTACTGGCA TCCCTGTTGC TAGTACGTTC ATGGGACTTG
961 GATCGTATCC TTGTGACGAT GAAGAGTTCT CTCTGCAAAT GCTAGGAATG CATGGAACAG

1021 TGTACGCTAA TTACGCTGTC GAGTATAGCG ATCTTCTGCT TGCTTTTGGG GTTAGGTTTG
1081 ACGACCGTGT GACCGGAAAG CTTGAGGCCT TTGCTAGCCG GGCCAAGATC GTGCATATTG
1141 ATATTGATTC TACCGAAATC GGGAAGAACA AGACACCTCA TGTGTCGGTG TGTTGTGATG
1201 TTCAGCTAGC CTTGCAAGGG ATGAACGAGG TTCTTGAGAA CCGACGAGAT GTGCTTGACT
1261 TCGGGGAATG GAGATGTGAA TTGAACGAAC AGAGACTAAA GTTCCCTCTC CGCTACAAGA
1321 CGTTTGGGGA AGAGATTCCT CCACAGTACG CCATTCAACT ACTTGACGAG CTAACCGACG
1381 GGAAGGCAAT TATCACTACT GGTGTCGGGC AACACCAGAT GTGGGCCGCC CAATTCTACA
1441 GATTCAAGAA ACCCCGCCAA TGGCTGTCTT CAGGAGGCCT AGGAGCCATG GGGTTCGGTC
1501 TTCCTGCAGC CATGGGAGCC GCTATAGCCA ACCCGGGAGC AGTGGTTGTC GACATTGATG
1561 GGGATGGTAG CTTCATCATG AACATTCAAG AACTGGCAAC CATCAGGGTT GAGAATCTTC
1621 CAGTCAAGGT TTTGCTGATT AATAATCAGC ACCTCGGAAT GGTCCTTCAG TGGGAAGACC
1681 ACTTCTACGC AGCTAACAGA GCCGATTCTT TTCTGGGAGA CCCGGCGAAC CCTGAGGCGG
1741 TATTCCCGGA TATGCTGTTG TTCGCCGCAT CGTGCGGTAT ACCAGCCGCC AGGGTCACCA
1801 GAAGGGAGGA CCTCCGAGAG GCAATCCAGA CAATGCTGGA CACACCTGGA CCATTCTTGT
1861 TGGATGTGGT CTGTCCTCAC CAGGACCATG TGTTACCACT CATCCCTAGT GGCGGCACCT
1921 TCAAGGACAT TATTGTGTAG TACTACTTTA ATGTTTGATT ATTCTATGCT CTCTTCTG
