# Supplementary material for: Genetic Origin of AHAS2 Genes in Brassica Allotetraploids and Association of Its Orthologs with Agronomic Traits in B. napus
Source: Plants (Basel). 2026 Apr 7;15(7):1126. doi: 10.3390/plants15071126 (PMC13074368; doi:10.3390/plants15071126)
Supplement: Supplementary file 1 [file plants-15-01126-s001.zip › Supplementary file S2.pdf]

Supplementary file 2. DNA sequences of *Bol.AHAS2* and orthologs in *Brassica* species

2E71

SEQ DNAMAN1: 2251 bp;

Composition 559 A; 577 C; 545 G; 570 T; 0 OTHER

Percentage: 25% A; 26% C; 24% G; 25% T; 0%OTHER

Molecular Weight (kDa): ssDNA: 694.31 dsDNA: 1387.7

ORIGIN

1 GTTGGTAGGT AAGATATCAT TAGCAAGGTG ATGTTAGGCG TATGTACTCT TTTTATACCA

61 TGGACCCTAG CTATTTGCAT GTATAAATTT TAGTTCTCTC TCACAGAGTT TATTTACTCA

121 TTCAAATTAA GCATTTCTCA CACTCTATTT TGCAAAATTA TATCTCCTCC TCTTACCCCC

181 ATGGCTTCGT CTTCGTTCTT CGGCACCATT ACGTCTTCTC CGACAAAAGC TTCCGCCTTC

241 TCCCTACCGG TGTGGGTAAC TACGCTCCCG TCCTTCCCGC GCCGCCGTGC TACTCGTGTC

301 TCCGTTTCGG CCAACTCGAA GAAAGACCAA GACCGCACAG CTTCACGTCG AGAGAATCCG

361 AGCACATTCA GCTCCAAATA CGCTCCCAAC GAGCCCCACA GTGGCGCAGA CATCCTGGTC

421 GAAGCCCTGG AGCGTCAAGG AGTGGACGTA GTCTTCGCTT ACCCAGGAGG CGCATCAATG

481 GAGATCCATC AAGCCCTAAC TTGCTCCAAC ACAATCCGAA ACGTCCTTCC CCGTCACGAA

541 CAAGGAGGAA TCTTCGCCGC CGAGGGTTAC GCTCGTTCCT CCGGTAAACC CGGAATCTGC

601 ATCGCCACTT CCGGTCCAGG AGCTATGAAT CTCGTCACCG GATTAGCCGA CGCCATTTTT

661 GACAGCGTAC CCCTCATCGC AATCACAGGA CAGGTCCGTC GCCGGATGAT TGGTACCATG

721 GCGTTCCAGG AGACACCCGT TGTTGAGGTA ACGAGGACTA TAATGAAACA TAACTATATT

781 GTTATGGAAG TTGAAGATAT ACCTAGGATC GTGCAAGAAG CTTTTTTCCT AGCAACTTCC

841 GTTAGGCCGG GACCGGTTCT TATCGACGTC CCCAAAGATG TTCAGCAACA GTTTGCGATT

901 CCTAACTGGG AACAGCCTAT GCGCTTACCT CTTTACATGT CTACGATGCC TAAACCCCCC

961 AAAGTTTCTC ACTTAGAGCA GATTCTTAGG TTGGTTTCGG AGTCTAAGAG GCCAGTCTTG

1021 TACGTTGGAG GTGGTTGTGT GAACTCGAGT GAGGAACTGC GCAGATTTGT GGAACTTACT

1081 GGCATCCCTG TTGCGAGTAC GTTCATGGGC CTTGGATCTT ATCCTTGTAA CGATGAAGAG
1141 TTCTCTCTGC AAATGCTAGG AATGCATGGA ACAGTGTACG CTAATTACGC TGTCGAGTAT
1201 AGCGATCTTC TGCTTGCTTT TGGCCTTAGG TTTGACGACC GTGTGACCGG AAAGCTTGAG
1261 GCCTTTGCTA GCCGGGCCAA GATCGTGCAC ATTGACATCG ATTCTACCGA AATCGGGAAG
1321 AACAAGACAC CTCACGTGTC GGTGTGTTGT GATGTTCAGC TAGCCTTGCA AGGGATGAAC
1381 GAGGTTCTTG AGAACCGACG AGATGTGCTT GACTTCGGTG ATTGGAGAGG TGAATTGAAC
1441 GAACAGAGAC TAAAGTTCCC TCTCCGCTTC AAGACGTTTG GGGAAGAGAT TCCTCCACAG
1501 TACGCCATTC AACTACTTGA CGAGCTAACC GACGGGAAGG CAATTATCAC TACTGGTGTC
1561 GGCCAACACC AGATGTGGGC CGCCCAATTC TACAGATTCA AGAAACCCCG CCAATGGCTG
1621 TCTTCAGGAG GCCAAGGAGC CATGGGGTTC GGTCTTCCTG CAGCCATGGG AGCCGCTATA
1681 GCCAACCCGG GAGCAGTGGT TGTCGACATT GATGGGGATG GTAGCTTTAT CATGAACATT
1741 CAAGAGCTGG CAACCATCAG GGTTGAAAAT CTCCCAGTCA AGGTTTTGCT GATTAACAAT
1801 CAACACCTCG GAATGGTCCT CCAGTGGGAG GACCACTTCT ACGCCGCTAA CCGAGCCGAT
1861 TCTTTTCTGG GAGACCCGGC GAACCCAGAG GCGGTATTCC CGGATATGCT GTTGTTCGCC
1921 GCATCGTGCG GTATACCAGC CGCCAGGGTC ACCAGAAGGG AGGACCTCCG AGAGGCAATC
1981 CAGACGATGC TGGACACACC TGGACCATTC TTGTTGGATG TGGTCTGTCC TCACCAGGAC
2041 CATGTGTTAC CACTCATCCC TAGTGGCGGC ACCTTCAAGG ACATTATTGC GTAGTACCGA
2101 ACCGAACCTA ACCGAAATAG ACAATATGGT TTGGTTTTGG TATATACCAT ATAAACCGAA
2161 TGGATATAAT TTTATAAAAA CCGTAGGATT TGGATATGGT TTGGTATATA ACCGAATAAA
2221 CCGAACAAAA CCGACTAAAA GTAGGAACAT G

2E72

SEQ DNAMAN2: 2251 bp;

Composition 559 A; 577 C; 545 G; 570 T; 0 OTHER

Percentage: 25% A; 26% C; 24% G; 25% T; 0%OTHER

Molecular Weight (kDa): ssDNA: 694.31 dsDNA: 1387.7

ORIGIN

1 GTTGGTAGGT AAGATATCAT TAGCAAGGTG ATGTTAGGCG TATGTACTCT TTTTATACCA

61 TGGACCCTAG CTATTTGCAT GTATAAATTT TAGTTCTCTC TCACAGAGTT TATTTACTCA

121 TTCAAATTAA GCATTTCTCA CACTCTATTT TGCAAAATTA TATCTCCTCC TCTTACCCCC

181 ATGGCTTCGT CTTCGTTCTT CGGCACCATT ACGTCTTCTC CGACAAAAGC TTCCGCCTTC

241 TCCCTACCGG TGTGGGTAAC TACGCTCCCG TCCTTCCCGC GCCGCCGTGC TACTCGTGTC

301 TCCGTTTCGG CCAACTCGAA GAAAGACCAA GACCGCACAG CTTCACGTCG AGAGAATCCG

361 AGCACATTCA GCTCCAAATA CGCTCCCAAC GAGCCCCACA GTGGCGCAGA CATCCTGGTC

421 GAAGCCCTGG AGCGTCAAGG AGTGGACGTA GTCTTCGCTT ACCCAGGAGG CGCATCAATG

481 GAGATCCATC AAGCCCTAAC TTGCTCCAAC ACAATCCGAA ACGTCCTTCC CCGTCACGAA

541 CAAGGAGGAA TCTTCGCCGC CGAGGGTTAC GCTCGTTCCT CCGGTAAACC CGGAATCTGC

601 ATCGCCACTT CCGGTCCAGG AGCTATGAAT CTCGTCACCG GATTAGCCGA CGCCATTTTT

661 GACAGCGTAC CCCTCATCGC AATCACAGGA CAGGTCCGTC GCCGGATGAT TGGTACCATG

721 GCGTTCCAGG AGACACCCGT TGTTGAGGTA ACGAGGACTA TAATGAAACA TAACTATATT

781 GTTATGGAAG TTGAAGATAT ACCTAGGATC GTGCAAGAAG CTTTTTTCCT AGCAACTTCC

841 GTTAGGCCGG GACCGGTTCT TATCGACGTC CCCAAAGATG TTCAGCAACA GTTTGCGATT

901 CCTAACTGGG AACAGCCTAT GCGCTTACCT CTTTACATGT CTACGATGCC TAAACCCCCC

961 AAAGTTTCTC ACTTAGAGCA GATTCTTAGG TTGGTTTCGG AGTCTAAGAG GCCAGTCTTG

1021 TACGTTGGAG GTGGTTGTGT GAACTCGAGT GAGGAACTGC GCAGATTTGT GGAACTTACT

1081 GGCATCCCTG TTGCGAGTAC GTTCATGGGC CTTGGATCTT ATCCTTGTAA CGATGAAGAG

1141 TTCTCTCTGC AAATGCTAGG AATGCATGGA ACAGTGTACG CTAATTACGC TGTCGAGTAT

1201 AGCGATCTTC TGCTTGCTTT TGGCCTTAGG TTTGACGACC GTGTGACCGG AAAGCTTGAG

1261 GCCTTTGCTA GCCGGGCCAA GATCGTGCAC ATTGACATCG ATTCTACCGA AATCGGGAAG
1321 AACAAGACAC CTCACGTGTC GGTGTGTTGT GATGTTCAGC TAGCCTTGCA AGGGATGAAC
1381 GAGGTTCTTG AGAACCGACG AGATGTGCTT GACTTCGGTG ATTGGAGAGG TGAATTGAAC
1441 GAACAGAGAC TAAAGTTCCC TCTCCGCTTC AAGACGTTTG GGGAAGAGAT TCCTCCACAG
1501 TACGCCATTC AACTACTTGA CGAGCTAACC GACGGGAAGG CAATTATCAC TACTGGTGTC
1561 GGCCAACACC AGATGTGGGC CGCCCAATTC TACAGATTCA AGAAACCCCG CCAATGGCTG
1621 TCTTCAGGAG GCCAAGGAGC CATGGGGTTC GGTCTTCCTG CAGCCATGGG AGCCGCTATA
1681 GCCAACCCGG GAGCAGTGGT TGTCGACATT GATGGGGATG GTAGCTTTAT CATGAACATT
1741 CAAGAGCTGG CAACCATCAG GGTTGAAAAT CTCCCAGTCA AGGTTTTGCT GATTAACAAT
1801 CAACACCTCG GAATGGTCCT CCAGTGGGAG GACCACTTCT ACGCCGCTAA CCGAGCCGAT
1861 TCTTTTCTGG GAGACCCGGC GAACCCAGAG GCGGTATTCC CGGATATGCT GTTGTTCGCC
1921 GCATCGTGCG GTATACCAGC CGCCAGGGTC ACCAGAAGGG AGGACCTCCG AGAGGCAATC
1981 CAGACGATGC TGGACACACC TGGACCATTC TTGTTGGATG TGGTCTGTCC TCACCAGGAC
2041 CATGTGTTAC CACTCATCCC TAGTGGCGGC ACCTTCAAGG ACATTATTGC GTAGTACCGA
2101 ACCGAACCTA ACCGAAATAG ACAATATGGT TTGGTTTTGG TATATACCAT ATAAACCGAA
2161 TGGATATAAT TTTATAAAAA CCGTAGGATT TGGATATGGT TTGGTATATA ACCGAATAAA
2221 CCGAACAAAA CCGACTAAAA GTAGGAACAT G

2E35

SEQ DNAMAN3: 2251 bp;

Composition 559 A; 577 C; 545 G; 570 T; 0 OTHER

Percentage: 25% A; 26% C; 24% G; 25% T; 0%OTHER

Molecular Weight (kDa): ssDNA: 694.31 dsDNA: 1387.7

ORIGIN

1 GTTGGTAGGT AAGATATCAT TAGCAAGGTG ATGTTAGGCG TATGTACTCT TTTTATACCA
61 TGGACCCTAG CTATTTGCAT GTATAAATTT TAGTTCTCTC TCACAGAGTT TATTTACTCA

121 TTCAAATTAA GCATTTCTCA CACTCTATTT TGCAAAATTA TATCTCCTCC TCTTACCCCC
181 ATGGCTTCGT CTTCGTTCTT CGGCACCATT ACGTCTTCTC CGACAAAAGC TTCCGCCTTC
241 TCCCTACCGG TGTGGGTAAC TACGCTCCCG TCCTTCCCGC GCCGCCGTGC TACTCGTGTC
301 TCCGTTTCGG CCAACTCGAA GAAAGACCAA GACCGCACAG CTTCACGTCG AGAGAATCCG
361 AGCACATTCA GCTCCAAATA CGCTCCCAAC GAGCCCCACA GTGGCGCAGA CATCCTGGTC
421 GAAGCCCTGG AGCGTCAAGG AGTGGACGTA GTCTTCGCTT ACCCAGGAGG CGCATCAATG
481 GAGATCCATC AAGCCCTAAC TTGCTCCAAC ACAATCCGAA ACGTCCTTCC CCGTCACGAA
541 CAAGGAGGAA TCTTCGCCGC CGAGGGTTAC GCTCGTTCCT CCGGTAAACC CGGAATCTGC
601 ATCGCCACTT CCGGTCCAGG AGCTATGAAT CTCGTCACCG GATTAGCCGA CGCCATTTTT
661 GACAGCGTAC CCCTCATCGC AATCACAGGA CAGGTCCGTC GCCGGATGAT TGGTACCATG
721 GCGTTCCAGG AGACACCCGT TGTTGAGGTA ACGAGGACTA TAATGAAACA TAACTATATT
781 GTTATGGAAG TTGAAGATAT ACCTAGGATC GTGCAAGAAG CTTTTTTCCT AGCAACTTCC
841 GTTAGGCCGG GACCGGTTCT TATCGACGTC CCCAAAGATG TTCAGCAACA GTTTGCGATT
901 CCTAACTGGG AACAGCCTAT GCGCTTACCT CTTTACATGT CTACGATGCC TAAACCCCCC
961 AAAGTTTCTC ACTTAGAGCA GATTCTTAGG TTGGTTTCGG AGTCTAAGAG GCCAGTCTTG
1021 TACGTTGGAG GTGGTTGTGT GAACTCGAGT GAGGAACTGC GCAGATTTGT GGAACTTACT
1081 GGCATCCCTG TTGCGAGTAC GTTCATGGGC CTTGGATCTT ATCCTTGTAA CGATGAAGAG
1141 TTCTCTCTGC AAATGCTAGG AATGCATGGA ACAGTGTACG CTAATTACGC TGTCGAGTAT
1201 AGCGATCTTC TGCTTGCTTT TGGCCTTAGG TTTGACGACC GTGTGACCGG AAAGCTTGAG
1261 GCCTTTGCTA GCCGGGCCAA GATCGTGCAC ATTGACATCG ATTCTACCGA AATCGGGAAG
1321 AACAAGACAC CTCACGTGTC GGTGTGTTGT GATGTTCAGC TAGCCTTGCA AGGGATGAAC
1381 GAGGTTCTTG AGAACCGACG AGATGTGCTT GACTTCGGTG ATTGGAGAGG TGAATTGAAC

1441 GAACAGAGAC TAAAGTTCCC TCTCCGCTTC AAGACGTTTG GGGAAGAGAT TCCTCCACAG
1501 TACGCCATTC AACTACTTGA CGAGCTAACC GACGGGAAGG CAATTATCAC TACTGGTGTC
1561 GGCCAACACC AGATGTGGGC CGCCCAATTC TACAGATTCA AGAAACCCCG CCAATGGCTG
1621 TCTTCAGGAG GCCAAGGAGC CATGGGGTTC GGTCTTCCTG CAGCCATGGG AGCCGCTATA
1681 GCCAACCCGG GAGCAGTGGT TGTCGACATT GATGGGGATG GTAGCTTTAT CATGAACATT
1741 CAAGAGCTGG CAACCATCAG GGTTGAAAAT CTCCCAGTCA AGGTTTTGCT GATTAACAAT
1801 CAACACCTCG GAATGGTCCT CCAGTGGGAG GACCACTTCT ACGCCGCTAA CCGAGCCGAT
1861 TCTTTTCTGG GAGACCCGGC GAACCCAGAG GCGGTATTCC CGGATATGCT GTTGTTCGCC
1921 GCATCGTGCG GTATACCAGC CGCCAGGGTC ACCAGAAGGG AGGACCTCCG AGAGGCAATC
1981 CAGACGATGC TGGACACACC TGGACCATTC TTGTTGGATG TGGTCTGTCC TCACCAGGAC
2041 CATGTGTTAC CACTCATCCC TAGTGGCGGC ACCTTCAAGG ACATTATTGC GTAGTACCGA
2101 ACCGAACCTA ACCGAAATAG ACAATATGGT TTGGTTTTGG TATATACCAT ATAAACCGAA
2161 TGGATATAAT TTTATAAAAA CCGTAGGATT TGGATATGGT TTGGTATATA ACCGAATAAA
2221 CCGAACAAAA CCGACTAAAA GTAGGAACAT G

2E36

SEQ DNAMAN4: 2251 bp;

Composition 559 A; 577 C; 545 G; 570 T; 0 OTHER

Percentage: 25% A; 26% C; 24% G; 25% T; 0%OTHER

Molecular Weight (kDa): ssDNA: 694.31 dsDNA: 1387.7

ORIGIN

1 GTTGGTAGGT AAGATATCAT TAGCAAGGTG ATGTTAGGCG TATGTACTCT TTTTATACCA
61 TGGACCCTAG CTATTTGCAT GTATAAATTT TAGTTCTCTC TCACAGAGTT TATTTACTCA
121 TTCAAATTAA GCATTTCTCA CACTCTATTT TGCAAAATTA TATCTCCTCC TCTTACCCCC
181 ATGGCTTCGT CTTCGTTCTT CGGCACCATT ACGTCTTCTC CGACAAAAGC TTCCGCCTTC
241 TCCCTACCGG TGTGGGTAAC TACGCTCCCG TCCTTCCCGC GCCGCCGTGC TACTCGTGTC

301 TCCGTTTCGG CCAACTCGAA GAAAGACCAA GACCGCACAG CTTCACGTCG AGAGAATCCG

361 AGCACATTCA GCTCCAAATA CGCTCCCAAC GAGCCCCACA GTGGCGCAGA CATCCTGGTC

421 GAAGCCCTGG AGCGTCAAGG AGTGGACGTA GTCTTCGCTT ACCCAGGAGG CGCATCAATG

481 GAGATCCATC AAGCCCTAAC TTGCTCCAAC ACAATCCGAA ACGTCCTTCC CCGTCACGAA

541 CAAGGAGGAA TCTTCGCCGC CGAGGGTTAC GCTCGTTCCT CCGGTAAACC CGGAATCTGC

601 ATCGCCACTT CCGGTCCAGG AGCTATGAAT CTCGTCACCG GATTAGCCGA CGCCATTTTT

661 GACAGCGTAC CCCTCATCGC AATCACAGGA CAGGTCCGTC GCCGGATGAT TGGTACCATG

721 GCGTTCCAGG AGACACCCGT TGTTGAGGTA ACGAGGACTA TAATGAAACA TAACTATATT

781 GTTATGGAAG TTGAAGATAT ACCTAGGATC GTGCAAGAAG CTTTTTTCCT AGCAACTTCC

841 GTTAGGCCGG GACCGGTTCT TATCGACGTC CCCAAAGATG TTCAGCAACA GTTTGCGATT

901 CCTAACTGGG AACAGCCTAT GCGCTTACCT CTTTACATGT CTACGATGCC TAAACCCCCC

961 AAAGTTTCTC ACTTAGAGCA GATTCTTAGG TTGGTTTCGG AGTCTAAGAG GCCAGTCTTG

1021 TACGTTGGAG GTGGTTGTGT GAACTCGAGT GAGGAACTGC GCAGATTTGT GGAACTTACT

1081 GGCATCCCTG TTGCGAGTAC GTTCATGGGC CTTGGATCTT ATCCTTGTAA CGATGAAGAG

1141 TTCTCTCTGC AAATGCTAGG AATGCATGGA ACAGTGTACG CTAATTACGC TGTCGAGTAT

1201 AGCGATCTTC TGCTTGCTTT TGGCCTTAGG TTTGACGACC GTGTGACCGG AAAGCTTGAG

1261 GCCTTTGCTA GCCGGGCCAA GATCGTGCAC ATTGACATCG ATTCTACCGA AATCGGGAAG

1321 AACAAGACAC CTCACGTGTC GGTGTGTTGT GATGTTCAGC TAGCCTTGCA AGGGATGAAC

1381 GAGGTTCTTG AGAACCGACG AGATGTGCTT GACTTCGGTG ATTGGAGAGG TGAATTGAAC

1441 GAACAGAGAC TAAAGTTCCC TCTCCGCTTC AAGACGTTTG GGGAAGAGAT TCCTCCACAG

1501 TACGCCATTC AACTACTTGA CGAGCTAACC GACGGGAAGG CAATTATCAC TACTGGTGTC

1561 GGCCAACACC AGATGTGGGC CGCCCAATTC TACAGATTCA AGAAACCCCG CCAATGGCTG

1621 TCTTCAGGAG GCCAAGGAGC CATGGGGTTC GGTCTTCCTG CAGCCATGGG AGCCGCTATA
1681 GCCAACCCGG GAGCAGTGGT TGTCGACATT GATGGGGATG GTAGCTTTAT CATGAACATT
1741 CAAGAGCTGG CAACCATCAG GGTTGAAAAT CTCCCAGTCA AGGTTTTGCT GATTAACAAT
1801 CAACACCTCG GAATGGTCCT CCAGTGGGAG GACCACTTCT ACGCCGCTAA CCGAGCCGAT
1861 TCTTTTCTGG GAGACCCGGC GAACCCAGAG GCGGTATTCC CGGATATGCT GTTGTTCGCC
1921 GCATCGTGCG GTATACCAGC CGCCAGGGTC ACCAGAAGGG AGGACCTCCG AGAGGCAATC
1981 CAGACGATGC TGGACACACC TGGACCATTC TTGTTGGATG TGGTCTGTCC TCACCAGGAC
2041 CATGTGTTAC CACTCATCCC TAGTGGCGGC ACCTTCAAGG ACATTATTGC GTAGTACCGA
2101 ACCGAACCTA ACCGAAATAG ACAATATGGT TTGGTTTTGG TATATACCAT ATAAACCGAA
2161 TGGATATAAT TTTATAAAAA CCGTAGGATT TGGATATGGT TTGGTATATA ACCGAATAAA
2221 CCGAACAAAA CCGACTAAAA GTAGGAACAT G

Xiaguang

SEQ DNAMAN5: 2251 bp;
Composition 559 A; 576 C; 545 G; 571 T; 0 OTHER
Percentage: 25% A; 26% C; 24% G; 25% T; 0%OTHER
Molecular Weight (kDa): ssDNA: 694.32 dsDNA: 1387.7
ORIGIN
1 GTTGGTAGGT AAGATATCAT TAGCAAGGTG ATGTTAGGCG TATGTACTCT TTTTATACCA
61 TGGACCCTAG CTATTTGCAT GTATAAATTT TAGTTCTCTC TCACAGAGTT TATTTACTCA
121 TTCAAATTAA GCATTTCTCA CACTCTATTT TGCAAAATTA TATCTCCTCC TCTTACCCCC
181 ATGGCTTCGT CTTCGTTCTT CGGCACCATT ACGTCTTCTC CGACAAAAGC TTCCGCCTTC
241 TCCCTACCGG TGTGGGTAAC TACGCTCCCG TCCTTCCCGC GCCGCCGTGC TACTCGTGTC
301 TCCGTTTCGG CCAACTCGAA GAAAGACCAA GACCGCACAG CTTCACGTCG AGAGAATCCG
361 AGCACATTCA GCTCCAAATA CGCTCCCAAC GAGCCCCACA GTGGCGCAGA CATCCTGGTC
421 GAAGCCCTGG AGCGTCAAGG AGTGGACGTA GTCTTCGCTT ACCCAGGAGG CGCATCAATG

481 GAGATCCATC AAGCCCTAAC TTGCTCCAAC ACAATCCGAA ACGTCCTTCC CCGTCACGAA

541 CAAGGAGGAA TCTTCGCCGC CGAGGGTTAC GCTCGTTCCT CCGGTAAACC CGGAATCTGC

601 ATCGCCACTT CCGGTCCAGG AGCTATGAAT CTCGTCACCG GATTAGCCGA CGCCATTTTT

661 GACAGCGTAC CCCTCATCGC AATCACAGGA CAGGTCCGTC GCCGGATGAT TGGTACCATG

721 GCGTTCCAGG AGACACCTGT TGTTGAGGTA ACGAGGACTA TAATGAAACA TAACTATATT

781 GTTATGGAAG TTGAAGATAT ACCTAGGATC GTGCAAGAAG CTTTTTTCCT AGCAACTTCC

841 GTTAGGCCGG GACCGGTTCT TATCGACGTC CCCAAAGATG TTCAGCAACA GTTTGCGATT

901 CCTAACTGGG AACAGCCTAT GCGCTTACCT CTTTACATGT CTACGATGCC TAAACCCCCC

961 AAAGTTTCTC ACTTAGAGCA GATTCTTAGG TTGGTTTCGG AGTCTAAGAG GCCAGTCTTG

1021 TACGTTGGAG GTGGTTGTGT GAACTCGAGT GAGGAACTGC GCAGATTTGT GGAACTTACT

1081 GGCATCCCTG TTGCGAGTAC GTTCATGGGC CTTGGATCTT ATCCTTGTAA CGATGAAGAG

1141 TTCTCTCTGC AAATGCTAGG AATGCATGGA ACAGTGTACG CTAATTACGC TGTCGAGTAT

1201 AGCGATCTTC TGCTTGCTTT TGGCCTTAGG TTTGACGACC GTGTGACCGG AAAGCTTGAG

1261 GCCTTTGCTA GCCGGGCCAA GATCGTGCAC ATTGACATCG ATTCTACCGA AATCGGGAAG

1321 AACAAGACAC CTCACGTGTC GGTGTGTTGT GATGTTCAGC TAGCCTTGCA AGGGATGAAC

1381 GAGGTTCTTG AGAACCGACG AGATGTGCTT GACTTCGGTG ATTGGAGAGG TGAATTGAAC

1441 GAACAGAGAC TAAAGTTCCC TCTCCGCTTC AAGACGTTTG GGGAAGAGAT TCCTCCACAG

1501 TACGCCATTC AACTACTTGA CGAGCTAACC GACGGGAAGG CAATTATCAC TACTGGTGTC

1561 GGCCAACACC AGATGTGGGC CGCCCAATTC TACAGATTCA AGAAACCCCG CCAATGGCTG

1621 TCTTCAGGAG GCCAAGGAGC CATGGGGTTC GGTCTTCCTG CAGCCATGGG AGCCGCTATA

1681 GCCAACCCGG GAGCAGTGGT TGTCGACATT GATGGGGATG GTAGCTTTAT CATGAACATT

1741 CAAGAGCTGG CAACCATCAG GGTTGAAAAT CTCCCAGTCA AGGTTTTGCT GATTAACAAT

1801 CAACACCTCG GAATGGTCCT CCAGTGGGAG GACCACTTCT ACGCCGCTAA CCGAGCCGAT

1861 TCTTTTCTGG GAGACCCGGC GAACCCAGAG GCGGTATTCC CGGATATGCT GTTGTTCGCC

1921 GCATCGTGCG GTATACCAGC CGCCAGGGTC ACCAGAAGGG AGGACCTCCG AGAGGCAATC

1981 CAGACGATGC TGGACACACC TGGACCATTC TTGTTGGATG TGGTCTGTCC TCACCAGGAC

2041 CATGTGTTAC CACTCATCCC TAGTGGCGGC ACCTTCAAGG ACATTATTGC GTAGTACCGA

2101 ACCGAACCTA ACCGAAATAG ACAATATGGT TTGGTTTTGG TATATACCAT ATAAACCGAA

2161 TGGATATAAT TTTATAAAAA CCGTAGGATT TGGATATGGT TTGGTATATA ACCGAATAAA

2221 CCGAACAAAA CCGACTAAAA GTAGGAACAT G

Qiutian

SEQ DNAMAN6: 2251 bp;

Composition 559 A; 577 C; 545 G; 570 T; 0 OTHER

Percentage: 25% A; 26% C; 24% G; 25% T; 0%OTHER

Molecular Weight (kDa): ssDNA: 694.31 dsDNA: 1387.7

ORIGIN

1 GTTGGTAGGT AAGATATCAT TAGCAAGGTG ATGTTAGGCG TATGTACTCT TTTTATACCA

61 TGGACCCTAG CTATTTGCAT GTATAAATTT TAGTTCTCTC TCACAGAGTT TATTTACTCA

121 TTCAAATTAA GCATTTCTCA CACTCTATTT TGCAAAATTA TATCTCCTCC TCTTACCCCC

181 ATGGCTTCGT CTTCGTTCTT CGGCACCATT ACGTCTTCTC CGACAAAAGC TTCCGCCTTC

241 TCCCTACCGG TGTGGGTAAC TACGCTCCCG TCCTTCCCGC GCCGCCGTGC TACTCGTGTC

301 TCCGTTTCGG CCAACTCGAA GAAAGACCAA GACCGCACAG CTTCACGTCG AGAGAATCCG

361 AGCACATTCA GCTCCAAATA CGCTCCCAAC GAGCCCCACA GTGGCGCAGA CATCCTGGTC

421 GAAGCCCTGG AGCGTCAAGG AGTGGACGTA GTCTTCGCTT ACCCAGGAGG CGCATCAATG

481 GAGATCCATC AAGCCCTAAC TTGCTCCAAC ACAATCCGAA ACGTCCTTCC CCGTCACGAA

541 CAAGGAGGAA TCTTCGCCGC CGAGGGTTAC GCTCGTTCCT CCGGTAAACC CGGAATCTGC

601 ATCGCCACTT CCGGTCCAGG AGCTATGAAT CTCGTCACCG GATTAGCCGA CGCCATTTTT

661 GACAGCGTAC CCCTCATCGC AATCACAGGA CAGGTCCGTC GCCGGATGAT TGGTACCATG

721 GCGTTCCAGG AGACACCTGT TGTTGAGGTA ACGAGGACTA TAATGAAACA TAACTATATT

781 GTTATGGAAG TTGAAGATAT ACCTAGGATC GTGCAAGAAG CTTTTTTCCT AGCAACTTCC

841 GTTAGGCCGG GACCGGTTCT TATCGACGTC CCCAAAGATG TTCAGCAACA GTTTGCGATT

901 CCTAACTGGG AACAGCCTAT GCGCTTACCT CTTTACATGT CTACGATGCC TAAACCCCCC

961 AAAGTTTCTC ACTTAGAGCA GATTCTTAGG TTGGTTTCGG AGTCTAAGAG GCCAGTCTTG

1021 TACGTTGGAG GTGGTTGTGT GAACTCGAGT GAGGAACTGC GCAGATTTGT GGAACTTACT

1081 GGCATCCCTG TTGCGAGTAC GTTCATGGGC CTTGGATCTT ATCCTTGTAA CGATGAAGAG

1141 TTCTCTCTGC AAATGCTAGG AATGCATGGA ACAGTGTACG CTAATTACGC TGTCGAGTAT

1201 AGCGATCTTC TGCTTGCTTT TGGCCTTAGG TTTGACGACC GTGTGACCGG AAAGCTTGAG

1261 GCCTTTGCTA GCCGGGCCAA GATCGTGCAC ATTGACATCG ATTCTACCGA AATCGGGAAG

1321 AACAAGACAC CTCACGTGTC GGTGTGTTGT GATGTTCAGC TAGCCTTGCA AGGGATGAAC

1381 GAGGTTCTTG AGAACCGACG AGATGTGCTT GACTTCGGTG ATTGGAGAGG TGAATTGAAC

1441 GAACAGAGAC TAAAGTTCCC TCTCCGCTTC AAGACGTTTG GGGAAGAGAT TCCTCCACAG

1501 TACGCCATTC AACTACTTGA CGAGCTAACC GACGGGAAGG CAATTATCAC TACTGGTGTC

1561 GGCCAACACC AGATGTGGGC CGCCCAATTC TACAGATTCA AGAAACCCCG CCAATGGCTG

1621 TCTTCAGGAG GCCAAGGAGC CATGGGGTTC GGTCTTCCTG CAGCCATGGG AGCCGCTATA

1681 GCCAACCCGG GAGCAGTGGT TGTCGACATT GATGGGGATG GTAGCTTTAT CATGAACATT

1741 CAAGAGCTGG CAACCATCAG GGTTGAAAAT CTCCCAGTCA AGGTTTTGCT GATTAACAAT

1801 CAACACCTCG GAATGGTCCT CCAGTGGGAG GACCACTTCT ACGCCGCTAA CCGAGCCGAT

1861 TCTTTTCTGG GAGACCCGGC GAACCCAGAG GCGGTATTCC CGGATATGCT GTCGTTCGCC

1921 GCATCGTGCG GTATACCAGC CGCCAGGGTC ACCAGAAGGG AGGACCTCCG AGAGGCAATC

1981 CAGACGATGC TGGACACACC TGGACCATTC TTGTTGGATG TGGTCTGTCC TCACCAGGAC

2041 CATGTGTTAC CACTCATCCC TAGTGGCGGC ACCTTCAAGG ACATTATTGC GTAGTACCGA

2101 ACCGAACCTA ACCGAAATAG ACAATATGGT TTGGTTTTGG TATATACCAT ATAAACCGAA

2161 TGGATATAAT TTTATAAAAA CCGTAGGATT TGGATATGGT TTGGTATATA ACCGAATAAA

2221 CCGAACAAAA CCGACTAAAA GTAGGAACAT G
