# Supplementary material for: Genetic Origin of AHAS2 Genes in Brassica Allotetraploids and Association of Its Orthologs with Agronomic Traits in B. napus
Source: Plants (Basel). 2026 Apr 7;15(7):1126. doi: 10.3390/plants15071126 (PMC13074368; doi:10.3390/plants15071126)
Supplement: Supplementary file 1 [file plants-15-01126-s001.zip › Supplementary Table and Figures.pdf]

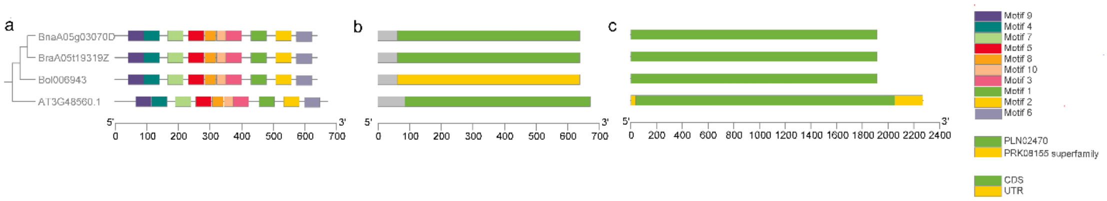

Figure S1. *AHAS2* gene structure and conserved domains of its encoded proteins. **(a)** Conserved motifs (motifs 1–10); **(b)** Conserved domains, PLN22470 and PRK08155 superfamily belong to the acetolactate synthase large subunit family; **(c)** Gene structure.

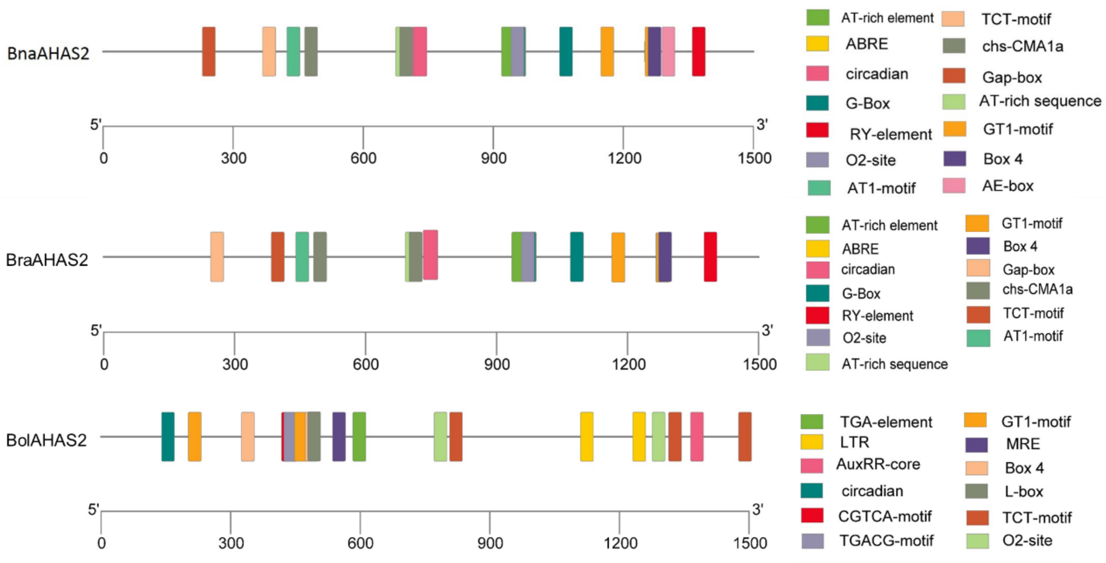

Figure S2. *Cis*-acting elements in the 1.5 kb upstream sequences of *Bra.AHAS2, Bna.AHAS2,* and *Bol.AHAS2*.

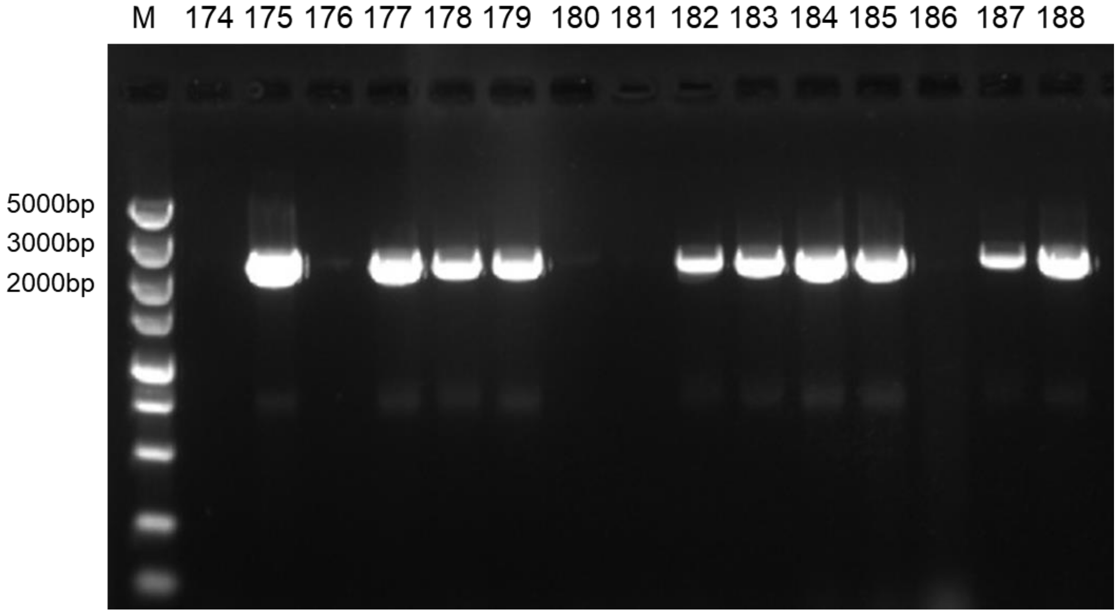

Figure S3. PCR amplification of *Bol.AHAS2* in *B. oleracea* (CC) accessions. M, DS5000 marker; the accession numbers 174~188 are shown in table S1.

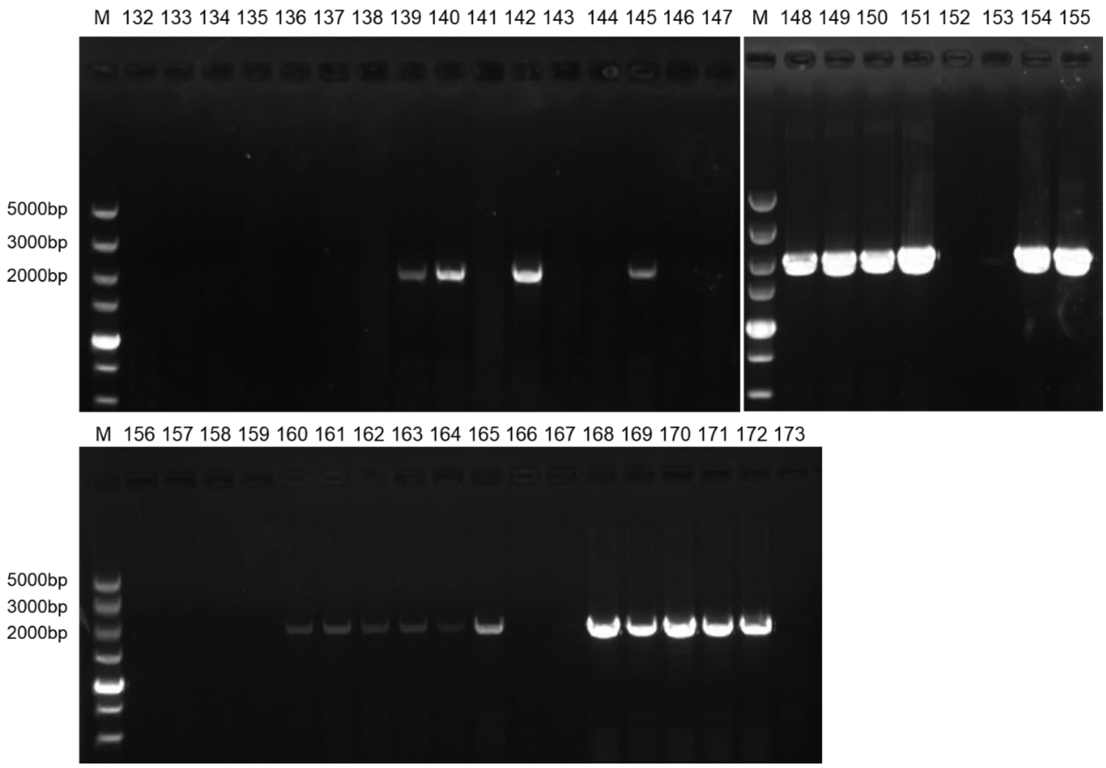

Figure S4. PCR amplification of *Bra.AHAS2* in *B. rapa* (AA) accessions. M, DS5000 marker; the accession numbers 132~173 are shown in table S1.

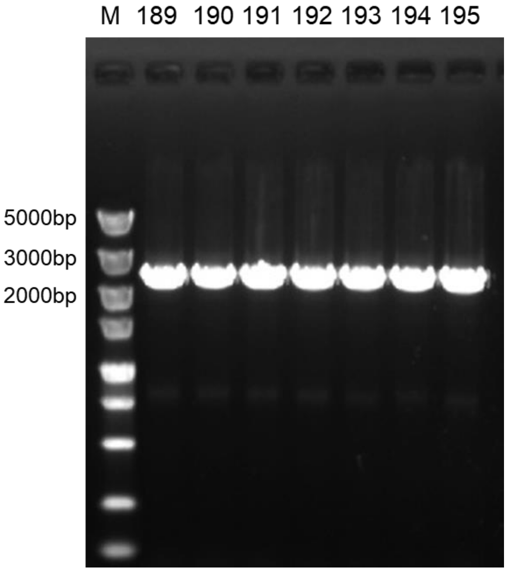

Figure S5. PCR amplification of *BcaC.AHAS2* in *B. carinata* (BBCC) accessions. M, DS5000 marker; the accession numbers 189~195 are shown in table S1.

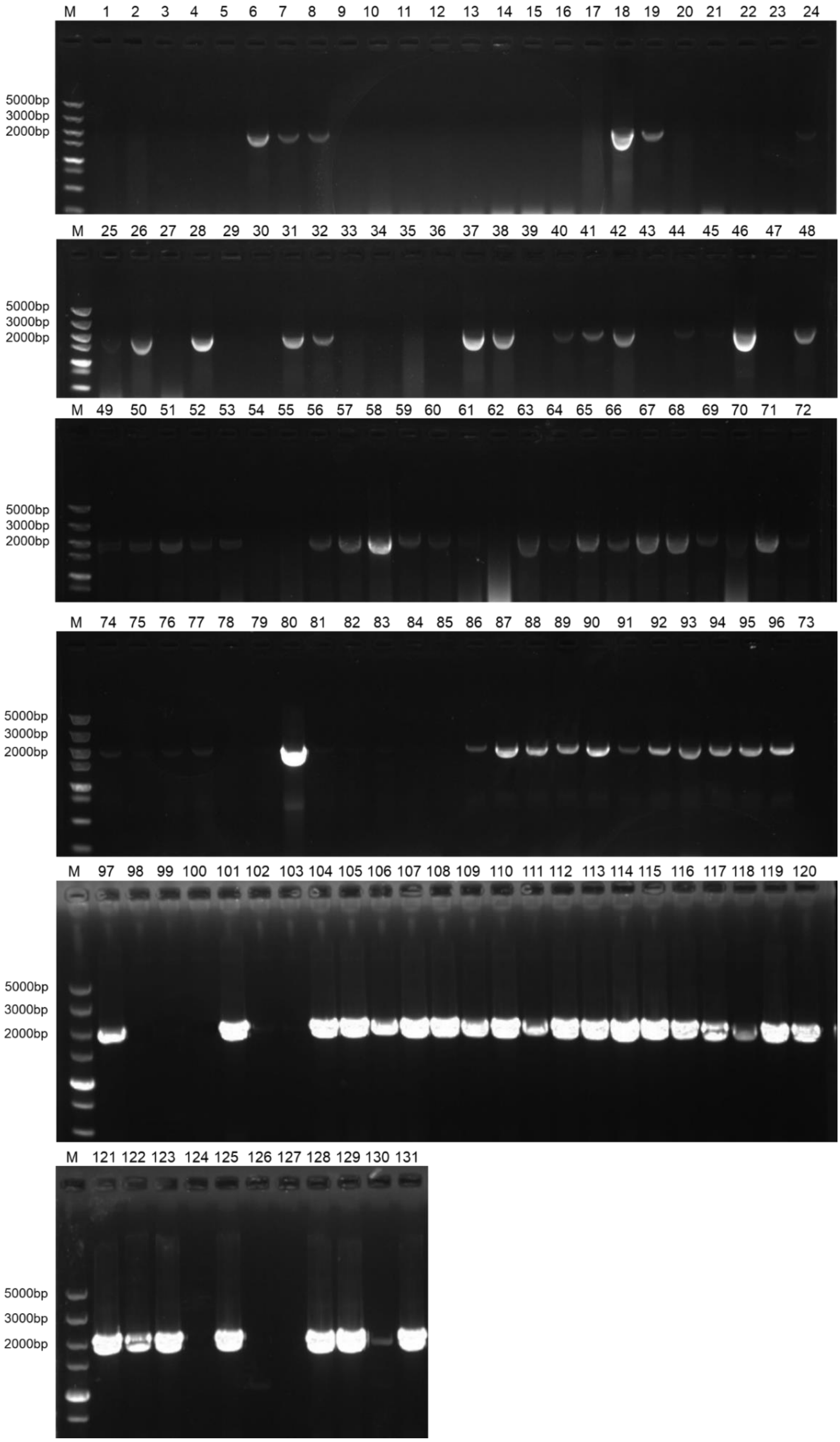

Figure S6. PCR amplification of *BnaA.AHAS2* in *B. napus* (AACC) accessions. M, DS5000 marker; the accession numbers 1~131 are shown in table S1.

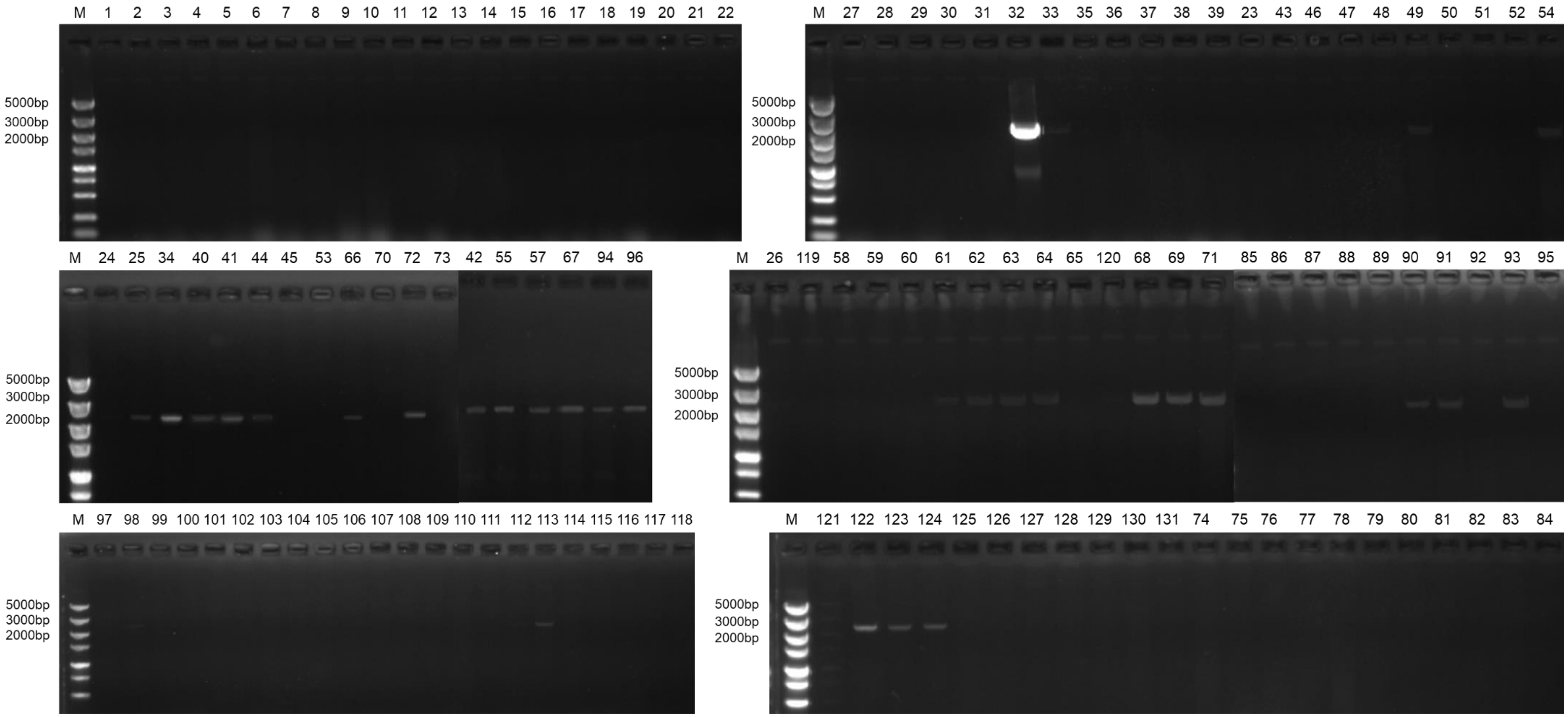

Figure S7. PCR amplification of *BnaC.AHAS2* in *B. napus* (AACC) accessions. M, DS5000 marker; the accession numbers 1~131 are shown in table S1.

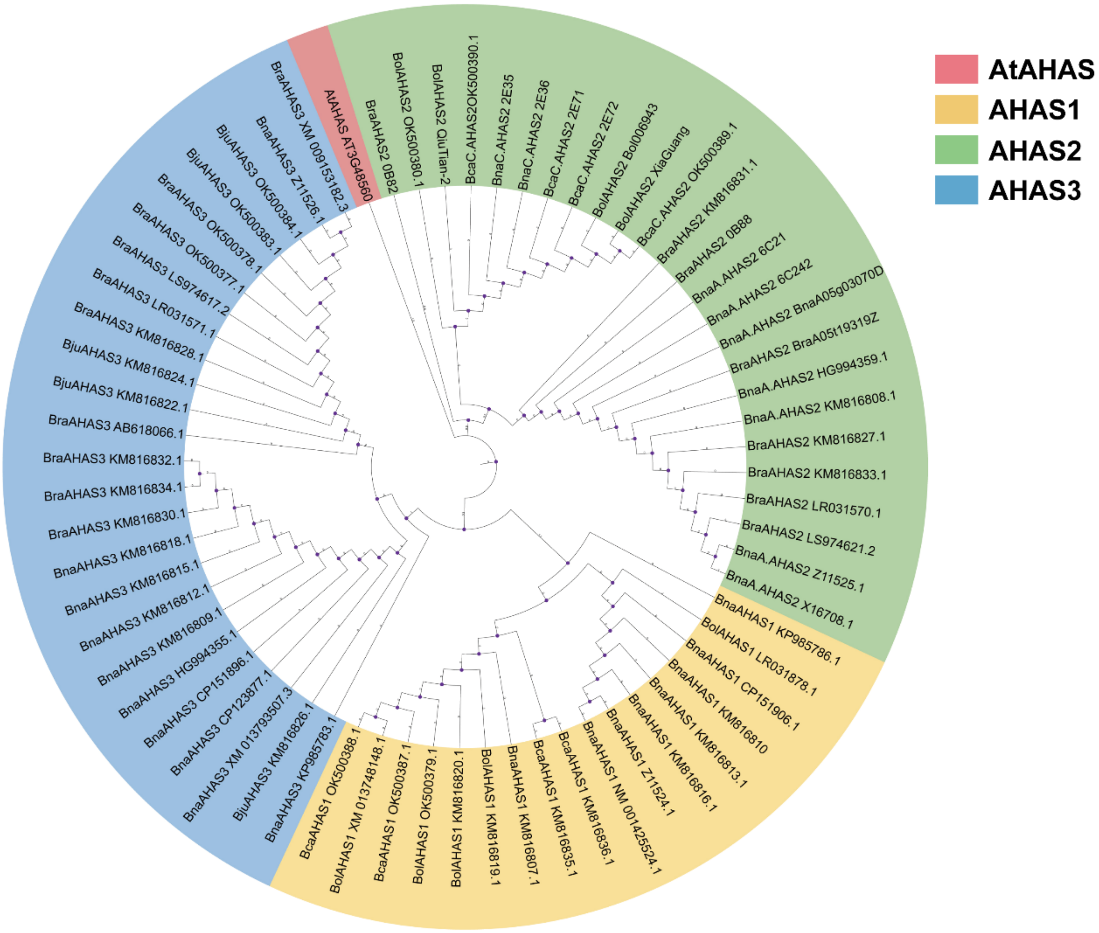

Figure S8. Phylogenetic analysis of AHAS2 in *Brassica* crops. All AHAS2 proteins are grouped into four branches, and each branch is represented by a different color. The red branch indicates that AHAS from *Arabidopsis thaliana*; the yellow branch indicates that AHAS1 from *Brassica*; the green branch indicates that AHAS2 from *Brassica*; the blue branch indicates that AHAS3 from *Brassica*. AtAHAS sequence from the TAIR database; sequences 0B82, 0B88, 6C21, 6C242, Qiutian-2, Xiaguang, 2E35, 2E36, 2E71, and 2E72 were obtained in the present study; other gene sequences from the NCBI database.

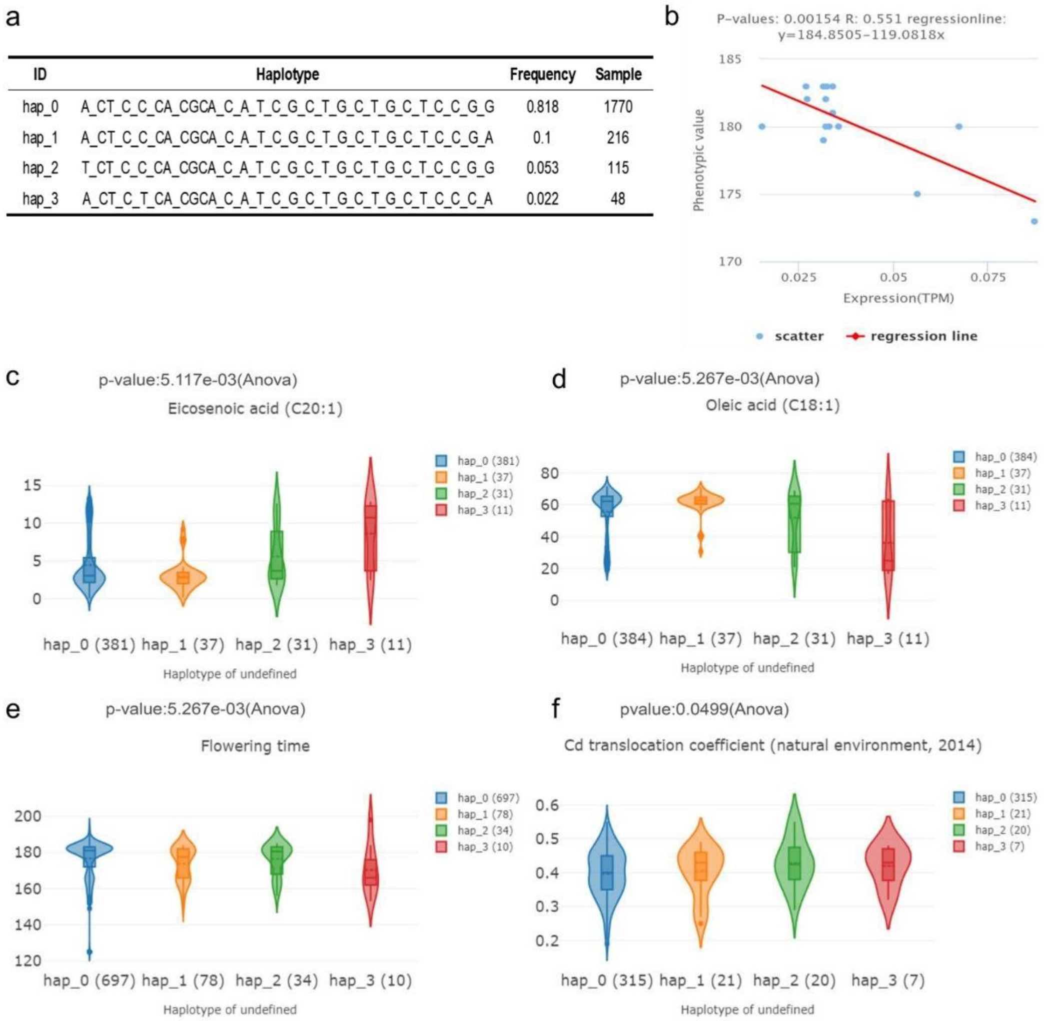

a

ID	Haplotype	Frequency	Sample
hap_0	A_CT_C_C_CA_CGCA_C_A_T_C_G_C_T_G_C_T_G_C_T_C_C_G_G	0.818	1770
hap_1	A_CT_C_C_CA_CGCA_C_A_T_C_G_C_T_G_C_T_G_C_T_C_C_G_A	0.1	216
hap_2	T_CT_C_C_CA_CGCA_C_A_T_C_G_C_T_G_C_T_G_C_T_C_C_G_G	0.053	115
hap_3	A_CT_C_T_CA_CGCA_C_A_T_C_G_C_T_G_C_T_G_C_T_C_C_C_A	0.022	48

Figure S9. *BnaA05g03070D* haplotype effect on associated traits and transcriptome-phenotype correlation analysis in *B. napus* accessions using the BnIR dataset. **(a)** Haplotype information of *BnaA05g03070D* and its ± 2 kb flanking region. **(b)** Correlation between *BnaA05g03070D* expression and flowering time. Haplotype effect of *BnaA05g03070D* on **(c)** flowering time, **(d)** oleic acid, **(e)** eicosenoic acid, and **(f)** cd translocation coefficient.

Table S1. Plant materials used in the present investigation.

No.	Code	Name	Type	*Bra.AHAS2 /BnaA.AHAS2*	*Bol.AHAS2 /BnaC.AHAS2 /BcaC.AHAS2*
1	1C04	HAU B-gan	*B. napus*	0	0
2	1C08	Zhongshuang 2S1	*B. napus*	0	0
3	1C15	Zhongshuang 2S2	*B. napus*	0	0
4	1C19	Zhongshuang 7-S1	*B. napus*	0	0
5	1C23	Zhongshuang 5	*B. napus*	0	0
6	1C25	Zhongshuang 9S1	*B. napus*	1	0
7	1C27	Zhongshuang 9S2	*B. napus*	1	0
8	1C29	Zhongshuang 9S3	*B. napus*	1	0
9	1C35	Zhongshuang 9III-1	*B. napus*	0	0
10	1C37	Zhongshuang 9III-2	*B. napus*	0	0
11	1C39	Zhongshuang 4	*B. napus*	0	0
12	1C41	CZ49-S1	*B. napus*	0	0
13	1C43	H15-S1	*B. napus*	0	0
14	1C47	ZS72	*B. napus*	0	0
15	1C49	ZY18-S1	*B. napus*	0	0
16	1C51	Shaan 2B	*B. napus*	0	0
17	1C53	2010B1-S1	*B. napus*	0	0
18	1C55	2010B6	*B. napus*	1	0
19	1C57	2010B7-S1	*B. napus*	1	0
20	1C61	2011B2-S1	*B. napus*	0	0
21	1C65	2011B2-S2	*B. napus*	0	0
22	1C67	2012B1-S1	*B. napus*	0	0
23	1C69	2012B1-S2	*B. napus*	0	0
24	1C71	2012B3	*B. napus*	0	0
25	1C77	2016B1	*B. napus*	0	1
26	1C84	2017B2	*B. napus*	1	0
27	1C90	2017B4	*B. napus*	0	0
28	1C95	2018B1	*B. napus*	1	0
29	1C102	2018B2	*B. napus*	0	0
30	1C120	2018B3	*B. napus*	0	0
31	1C131	2018B4	*B. napus*	1	0

No.	Code	Name	Type	*Bra.AHAS2 /BnaA.AHAS2*	*Bol.AHAS2 /BnaC.AHAS2 /BcaC.AHAS2*
32	1C138	2018B8	*B. napus*	1	1
33	1C167	2018B9	*B. napus*	0	0
34	1C179	2019B1	*B. napus*	0	1
35	1C196	2019B2-S1	*B. napus*	0	0
36	1C205	2020B2-S2	*B. napus*	0	0
37	1C213	2020B3	*B. napus*	1	0
38	1C215	2020B4	*B. napus*	1	0
39	1C218	2020B5	*B. napus*	0	0
40	1C227	2016 Zhong 9-TBM	*B. napus*	1	1
41	1C233	HAU-CR	*B. napus*	1	1
42	1C238	HAU-CR typeIII	*B. napus*	1	1
43	1C242	Huayou-2	*B. napus*	0	0
44	1C247	H9722	*B. napus*	0	1
45	1C248	H9722 Var1	*B. napus*	0	0
46	1C249	S8R	*B. napus*	1	0
47	1C254	QY211R	*B. napus*	0	0
48	1C259	Ningza1R	*B. napus*	1	0
49	1C264	Q86R	*B. napus*	1	1
50	1C267	SH11	*B. napus*	1	0
51	1C271	SH11za-1	*B. napus*	1	0
52	1C274	Y6-S1	*B. napus*	1	0
53	1C275	2000-5R-S1	*B. napus*	1	0
54	1C276	HYZ01R	*B. napus*	0	1
55	1C281	Zhong 821R-S1	*B. napus*	0	1
56	1C282	Z6C Var1	*B. napus*	1	0
57	1C283	Z6C-S1	*B. napus*	1	1
58	1C284	QSC-S1	*B. napus*	1	0
59	1C285	QSC-S2	*B. napus*	1	0
60	1C287	S9R	*B. napus*	1	0
61	1C288	CY1R	*B. napus*	0	1
62	1C290	S11R-S1	*B. napus*	0	1
63	1C293	Liu4	*B. napus*	1	1

No.	Code	Name	Type	*Bra.AHAS2 /BnaA.AHAS2*	*Bol.AHAS2 /BnaC.AHAS2 /BcaC.AHAS2*
64	1C294	Q7C	*B. napus*	1	1
65	1C297	Q7C-Var1	*B. napus*	1	0
66	1C298	Q10C-S1	*B. napus*	1	1
67	1C300	Q10C-S2	*B. napus*	1	1
68	1C301	Q10R	*B. napus*	1	1
69	1C305	D1526-S1	*B. napus*	1	1
70	1C306	CZL-20A	*B. napus*	0	0
71	1C309	CZL-2012	*B. napus*	1	1
72	1C311	CZL-20	*B. napus*	0	1
73	1C312	CH15R	*B. napus*	0	0
74	6C10	Zhongshuang 7-S2	*B. napus*	0	0
75	6C21	Zhongshuang 9S4	*B. napus*	1	0
76	6C47	Zhongshuang 9II	*B. napus*	1	0
77	6C64	CZ49-S2	*B. napus*	0	0
78	6C70	H15-S2	*B. napus*	0	0
79	6C76	New B1	*B. napus*	0	0
80	6C88	ZY18-S2	*B. napus*	0	0
81	6C96	2010B1-S2	*B. napus*	0	0
82	6C100	2010B4	*B. napus*	1	0
83	6C109	2010B7-S2	*B. napus*	1	0
84	6C124	2012B1-S3	*B. napus*	0	0
85	6C163	9722	*B. napus*	1	0
86	6C184	Qinyan 211R	*B. napus*	0	0
87	6C212	Y6-S2	*B. napus*	1	0
88	6C214	2000-5R-S2	*B. napus*	1	0
89	6C216	HYZ01R var1	*B. napus*	0	0
90	6C226	Zhong 821R-S2	*B. napus*	0	1
91	6C229	Z6C-S2	*B. napus*	1	1
92	6C230	QSC-S3	*B. napus*	1	0
93	6C239	S11R-S2	*B. napus*	0	1
94	6C242	Qin7C	*B. napus*	1	1
95	6C251	Q10C Var1	*B. napus*	1	0

No.	Code	Name	Type	*Bra.AHAS2 /BnaA.AHAS2*	*Bol.AHAS2 /BnaC.AHAS2 /BcaC.AHAS2*
96	6C260	D1526-S2	*B. napus*	1	1
97	2E10	Qin 3	*B. napus*	1	0
98	2E11	Westar	*B. napus*	0	0
99	2E12	Bronowski-S1	*B. napus*	0	0
100	2E13	Bronowski-S2	*B. napus*	0	0
101	2E14	Gan 1MP	*B. napus*	1	0
102	2E15	Zhongyou 821var1	*B. napus*	0	0
103	2E16	C3	*B. napus*	0	0
104	2E17	KS1701	*B. napus*	1	0
105	2E18	KS2002	*B. napus*	1	0
106	2E19	KS2185	*B. napus*	1	0
107	2E20	KS3017	*B. napus*	1	0
108	2E21	KS3068	*B. napus*	1	0
109	2E22	KS3073	*B. napus*	1	0
110	2E23	KS3074	*B. napus*	1	0
111	2E24	KS3077	*B. napus*	1	0
112	2E25	KS3132	*B. napus*	1	0
113	2E26	KS3248	*B. napus*	1	1
114	2E27	KS3254	*B. napus*	1	0
115	2E28	KS3302	*B. napus*	1	0
116	2E29	KS3350	*B. napus*	1	0
117	2E30	KS3357	*B. napus*	1	0
118	2E31	KS4022	*B. napus*	1	0
119	2E32	New B. napus	*B. napus*	1	0
120	2E33	Viking	*B. napus*	1	0
121	2E34	Profit	*B. napus*	1	0
122	2E35	Elect	*B. napus*	1	1
123	2E36	SP Arama	*B. napus*	1	1
124	2E37	82089	*B. napus*	0	1
125	2E38	Jetton	*B. napus*	1	0
126	2E39	Marathow	*B. napus*	0	0
127	2E40	Runba Var1	*B. napus*	0	0

No.	Code	Name	Type	*Bra.AHAS2 /BnaA.AHAS2*	*Bol.AHAS2 /BnaC.AHAS2 /BcaC.AHAS2*
128	2E41	Runba	*B. napus*	1	0
129	2E42	Shaan 2C	*B. napus*	1	0
130	2E43	Zhongyou 821	*B. napus*	1	0
131	2E44	Midas	*B. napus*	1	0
132	0B29	Cui lv80 tiancaixin	*B. rapa* subsp. *chinensis*	0	
133	0B30	60tian tiancaixin	*B. rapa* subsp. *chinensis*	0	
134	0B77	Huainan huangxincai	*B. rapa* subsp. *chinensis*	0	
135	0B78	Shanghaiqin	*B. rapa* subsp. *chinensis*	0	
136	0B79	Siji xiaobaicai	*B. rapa* subsp. *chinensis*	0	
137	0B80	Siji xiaobaicai	*B. rapa* subsp. *chinensis*	0	
138	0B81	Heiyoubaicai	*B. rapa* subsp. *chinensis*	0	
139	0B82	Tianyou-2	*B. rapa* subsp. *oleifera*	1	
140	0B83	Tianyou-8	*B. rapa* subsp. *oleifera*	1	
141	0B84	737	*B. rapa* subsp. *oleifera*	0	
142	0B85	737	*B. rapa* subsp. *oleifera*	1	
143	0B86	Longyou-6	*B. rapa* subsp. *oleifera*	0	
144	0B87	Brachina	*B. rapa* subsp. *oleifera*	0	
145	0B88	Evvisa	*B. rapa* subsp. *oleifera*	1	
146	0B89	Baiyu	*B. rapa* subsp. *oleifera*	0	
147	0B90	Nap CMS	*B. rapa* subsp. *oleifera*	0	
148	2E45	Siji xiaobaicai	*B. rapa* subsp. *chinensis*	1	
149	2E46	Longyou-6	*B. rapa* subsp. *oleifera*	1	

No.	Code	Name	Type	*Bra.AHAS2/BnaA.AHAS2*	*Bol.AHAS2/BnaC.AHAS2/BcaC.AHAS2*
150	2E47	Brachina	*B. rapa* subsp. *oleifera*	1	
151	2E48	Evvisa	*B. rapa* subsp. *oleifera*	1	
152	2E49	Baiyu	*B. rapa* subsp. *oleifera*	0	
153	2E50	Linyi xiaobaicai	*B. rapa* subsp. *chinensis*	0	
154	2E51	Tianyou-15	*B. rapa* subsp. *oleifera*	1	
155	2E52	Tobin	*B. rapa* subsp. *oleifera*	1	
156	2E53	721-1	*B. rapa* subsp. *oleifera*	0	
157	2E54	Baiye tacai	*B. rapa* subsp. *chinensis* var. *tai-tsai*	0	
158	2E55	Shanghai jimaocai	*B. rapa* subsp. *chinensis*	0	
159	2E56	Huainan huangxincai	*B. rapa* subsp. *chinensis*	0	
160	2E57	Rekang 80	*B. rapa* subsp. *pekinensis*	1	
161	2E58	Siji xiaobaicai	*B. rapa* subsp. *chinensis*	1	
162	2E59	Shanghaiqin	*B. rapa* subsp. *chinensis*	1	
163	2E60	Shanghaiqin yongan	*B. rapa* subsp. *chinensis*	1	
164	2E61	Heiyoubaicai	*B. rapa* subsp. *chinensis*	1	
165	2E62	Xialvmingxing	*B. rapa* subsp. *chinensis*	1	
166	2E63	Heiyoubaicai	*B. rapa* subsp. *chinensis*	0	
167	2E64	R500	*B. rapa* subsp. *triocularis*	0	
168	2E65	Zangyou-5	*B. rapa* subsp. *oleifera*	1	
169	2E66	Zangyou-3	*B. rapa* subsp. *oleifera*	1	

No.	Code	Name	Type	*Bra.AHAS2 /BnaA.AHAS2*	*Bol.AHAS2 /BnaC.AHAS2 /BcaC.AHAS2*
170	2E67	TR2	*B. rapa* subsp. *oleifera*	1	
171	2E68	AZ parkland	*B. rapa* subsp. *oleifera*	1	
172	2E69	Xichanghuangzi	*B. rapa* subsp. *oleifera*	1	
173	2E74	Sizaja	*B. rapa* subsp. *oleifera*	0	
174		Daguopingtou Ganlan	*B. oleracea* var. *capitata*		0
175		Qiutian No.2	*B. oleracea* var. *capitata*		1
176		Wanfeng	*B. oleracea* var. *capitata*		0
177		Baozi Ganlan	*B. oleracea* var. *gemmifera*		1
178		Yuyi ganlan F1	*B. oleracea* var. *acephala*		1
179		Beifangxiadi Ganlan	*B. oleracea* var. *capitata*		1
180		Jinyuanbao No.1	*B. oleracea* var. *capitata*		0
181		Jingfeng No.1	*B. oleracea* var. *capitata*		0
182		Chunfeng Ganlan	*B. oleracea* var. *capitata*		1
183		Xiaguang	*B. oleracea* var. *capitata*		1
184		Ribenzaosheng Ganlan	*B. oleracea* var. *capitata*		1
185		Jingxuan 8398	*B. oleracea* var. *capitata*		1
186		Helan 83	*B. oleracea* var. *capitata*		0
187		Tiancuishuiguo Ganlan	*B. oleracea* var. *capitata*		1
188	2E85	Texuan Zhonggan 11	*B. oleracea* var. *capitata*		1
189	2E71	CZ1404	*B. carinata*		1
190	2E72	CZ1402	*B. carinata*		1

No.	Code	Name	Type	*Bra.AHAS2 /BnaA.AHAS2*	*Bol.AHAS2 /BnaC.AHAS2 /BcaC.AHAS2*
191	2E75	Dodalla	*B. carinata*		1
192	2E76	CZ1401	*B. carinata*		1
193	2E77	BC-02	*B. carinata*		1
194	2E78	BC-04	*B. carinata*		1
195	2E79	150060008	*B. carinata*		1
196	2E80	Heijie	*B.nigra*	0	0
197	2E81	15006004	*B.nigra*	0	0
198	2E82	GBRC2933	*B.nigra*	0	0
199	1B03	Hengkoujie-4	*B. juncea* subsp. *integrifolia* var. *rugosa*	0	0
200	1B05	Hengkoujie-5	*B. juncea* subsp. *integrifolia* var. *rugosa*	0	0
201	1B07	Yangxianzhuyuanjie-1	*B. juncea* subsp. *integrifolia* var. *rugosa*	0	0
202	1B09	Mianxianjie-1	*B. juncea* subsp. *integrifolia* var. *rugosa*	0	0
203	1B12	Bendatoucai	*B. juncea* subsp. *napiformis* var. *megarrhiza*	0	0
204	1B16	HAU B-jiecai	*B. juncea* var.*gracilis*	0	0
205	1B19	Hanzhongjie	*B. juncea* var.*gracilis*	0	0
206	1B22	Hengkoujie-1	*B. juncea* subsp. *integrifolia* var. *rugosa*	0	0
207	1B23	Hengkoujie-2	*B. juncea* subsp. *integrifolia* var. *rugosa*	0	0
208	1B24	Hengkoujie-3	*B. juncea* subsp. *integrifolia* var. *rugosa*	0	0
209	1B27	Yangxianzhuyuanjie-1	*B. juncea* subsp. *integrifolia* var. *rugosa*	0	0

No.	Code	Name	Type	*Bra.AHAS2 /BnaA.AHAS2*	*Bol.AHAS2 /BnaC.AHAS2 /BcaC.AHAS2*
210	1B28	Mianxianjie-2	*B. juncea* subsp. *integrifolia* var. *rugosa*	0	0
211	1B29	Mianxianjie-3	*B. juncea* subsp. *integrifolia* var. *rugosa*	0	0
212	1B36	Quelv-2	*B. juncea* var.*gracilis*	0	0
213	1B37	Liujiecai	*B. juncea* subsp. *integrifolia* var. *strumata*	0	0
214	1B39	Dapinpubaoxinjiecai	*B. juncea* subsp. *integrifolia* var. *involuta*	0	0
215	1B41	Linongbaoxinjiecai	*B. juncea* subsp. *integrifolia* var. *involuta*	0	0
216	1B43	Tiancuidanainaicai	*B. juncea* subsp. *integrifolia* var. *strumata*	0	0
217	1B44	Zacai	*B. juncea* subsp. *tumida*	0	0
218	1B45	Zheyouzacai	*B. juncea* subsp. *tumida*	0	0
219	1B46	Beifangzacai	*B. juncea* subsp. *tumida*	0	0
220	1B48	Jinsijie	*B. juncea* subsp. *integrifolia* var. *multisect*	0	0
221	1B50	Jiutouniaoxuelihong	*B. juncea* subsp. *integrifolia* var. *multiceps*	0	0
222	1B51	Xiyexueuelihong	*B. juncea* subsp. *integrifolia* var. *multiceps*	0	0
223	1B53	Changfengguangtoujiecai	*B. juncea* subsp. *napiformis* var. *megarrhiza*	0	0
224	1B55	Ribenguangjie	*B. juncea* subsp. *napiformis* var. *megarrhiza*	0	0

No.	Code	Name	Type	*Bra.AHAS2 /BnaA.AHAS2*	*Bol.AHAS2 /BnaC.AHAS2 /BcaC.AHAS2*
225	1B57	Bendatoucai	*B. juncea* subsp. *napiformis* var. *megarrhiza*	0	0
226	1B58	Huangzijiecai	*B. juncea* var.*gracilis*	0	0
227	2E70	Hetianjiecai	*B. juncea* var.*gracilis*	0	0

Note: 1, amplified; 0, not amplified.

Table S2. Upstream *cis*-acting elements of *Bra.AHAS2*, *Bna.AHAS2*, and *Bol.AHAS2* genes.

Function	Promoter name	Promoter annotation	Gene		
			Bna.AHAS2	*Bol.AHAS2*	*Bra.AHAS2*
**Motifs related to light response**	G-Box	cis-acting regulatory element involved in light responsiveness	2	0	2
	GT1-motif	light responsive element	3	2	3
	MRE	MYB binding site involved in light responsiveness	0	1	0
	Box 4	part of a conserved DNA module involved in light responsiveness	1	2	1
	L-box	part of a light responsive element	0	1	0
	TCT-motif	part of a light responsive element	1	3	1
	Gap-box	part of a light responsive element	1	0	1
	chs-CMA1a	part of a light responsive element	2	0	2
	AT1-motif	part of a light responsive module	1	0	1
	AE-box	part of a light responsive module	1	0	0
**Motifs related to hormone response**	TGA-element	auxin-responsive element	0	1	0
	ABRE	cis-acting element involved in the abscisic acid responsiveness	1	0	1
	AuxRR-core	cis-acting regulatory element involved in auxin responsiveness	0	1	0
	CGTCA-motif	cis-acting regulatory element involved in the MeJA-responsiveness	0	1	0
	TGACG-motif	cis-acting regulatory element involved in the MeJA-responsiveness	0	1	0
**Motifs related to growth and development**	AT-rich element	binding site of AT-rich DNA binding protein (ATBP-1)	1	0	1
	circadian	cis-acting regulatory element involved in circadian control	2	1	2
	RY-element	cis-acting regulatory element involved in seed-specific regulation	1	0	1
	O2-site	cis-acting regulatory element involved in zein metabolism regulation	1	2	1
**Motif related to stress response**	LTR	cis-acting element involved in low-temperature responsiveness	0	2	0

Table S3. Comparison of DNA and protein sequences of *Bra.AHAS2* and its orthologs across *Brassica* species.

No.	Accession name	Type	Haplotype	Amplified product length (bp)	Coding sequence (bp)	The predicated proteins (aa)	SNP or InDel variation[1]		
							Indel (699 bp~707 bp)	SNP (1107 bp)	SNP (1625 bp)
1	Z11525.1	*B. napus*	Hap_0		1914	637	TGTTCAGCA (AspValGln)	C(Val)	A (His)
2	6C21	*B. napus*	Hap_1	1984	1914	637	TGTTCAGCA	G (Val)	A
3	6C242	*B. napus*	Hap_1	1984	1914	637	TGTTCAGCA	G	A
4	0B88	*B. rapa*	Hap_2	1984	1914	637	TGTTCAGCA	C	G (Arg)
5	0B82	*B. rapa*	Hap_3	1975	1905	634	deletion	G	A

Note: [1], The first base of the reference gene Z11525.1 start codon (ATG) is 1 bp. The letters in parentheses indicate the corresponding amino acids.

Table S4. Comparison of DNA and protein sequences of *Bol.AHAS2* and its orthologs across *Brassica* species.

No.	Accession name	Type	Haplotype	Amplified product length (bp)	Coding sequence (bp)	The predicated proteins (aa)	SNP[1]	
							558 bp	1733 bp
1	*Bol006943*	*B. oleracea*	Hap_0		1914	637	T (Pro)	T (Leu)
2	2E71	*B. carinata*	Hap_2	2251	1914	637	C (Pro)	T
3	2E72	*B. carinata*	Hap_2	2251	1914	637	C	T
4	2E35	*B. napus*	Hap_2	2251	1914	637	C	T
5	2E36	*B. napus*	Hap_2	2251	1914	637	C	T
6	Xiaguang	*B. oleracea*	Hap_0	2251	1914	637	T	T
7	Qiutian-2	*B. oleracea*	Hap_1	2251	1914	637	T	C (Ser)

Note: [1], The first base of the reference gene *Bol006943* start codon (ATG) is 1 bp. The letters in parentheses indicate the corresponding amino acids.

Table S5. Herbicide resistance of 131 *B. napus* accessions.

No.	Code	Name	Phytotoxicity Index	Inhibition ratio of Leave number	Inhibition ratio of leaf angle	Death rate	Average membership function Value
1	1C04	HAU B-gan	0.48	0.37	0.58	100	0.78
2	1C08	Zhongshuang 2S1	0.77	0.3	0.65	95.58	0.58
3	1C15	Zhongshuang 2S2	0.77	0.3	0.65	95.58	0.58
4	1C19	Zhongshuang 7-S1	0.5	0.27	0.75	97.97	0.75
5	1C23	Zhongshuang 5	0.48	0.37	0.58	100	0.78
6	1C25	Zhongshuang 9S1	0.42	0.34	0.63	99	0.82
7	1C27	Zhongshuang 9S2	0.42	0.34	0.6	99	0.82
8	1C29	Zhongshuang 9S3	0.52	0.29	0.41	100	0.6
9	1C35	Zhongshuang 9III-1	0.78	0.33	0.66	100	0.61
10	1C37	Zhongshuang 9III-2	0.78	0.33	0.61	100	0.61
11	1C39	Zhongshuang 4	0.56	0.23	0.46	98.54	0.54
12	1C41	CZ49-S1	0.87	0.3	0.63	71.26	0.23
13	1C43	H15-S1	0.81	0.27	0.68	100	0.59
14	1C47	ZS72	0.58	0.33	0.71	98.89	0.77
15	1C49	ZY18-S1	0.82	0.33	0.76	82.8	0.52
16	1C51	Shaan 2B	0.6	0.39	0.53	98.82	0.69
17	1C53	2010B1-S1	0.42	0.34	0.63	99	0.82
18	1C55	2010B6	0.47	0.37	0.71	98.96	0.88
19	1C57	2010B7-S1	0.47	0.37	0.74	98.96	0.88
20	1C61	2011B2-S1	0.5	0.39	0.71	92.47	0.81
21	1C65	2011B2-S2	0.5	0.27	0.7	97.97	0.75
22	1C67	2012B1-S1	0.51	0.33	0.45	100	0.6
23	1C69	2012B1-S2	0.52	0.29	0.49	100	0.6
24	1C71	2012B3	0.56	0.23	0.47	98.54	0.54
25	1C77	2016B1	0.6	0.39	0.53	98.82	0.69

26	1C84	2017B2	0.6	0.39	0.53	98.82	0.69
27	1C90	2017B4	0.65	0.21	0.58	89.65	0.47
28	1C95	2018B1	0.67	0.4	0.71	98.88	0.79
29	1C102	2018B2	0.67	0.4	0.74	98.88	0.79
30	1C120	2018B3	0.69	0.29	0.64	98.96	0.64
31	1C131	2018B4	0.69	0.29	0.64	98.96	0.64
32	1C138	2018B8	0.75	0.24	0.58	100	0.53
33	1C167	2018B9	0.77	0.3	0.65	95.58	0.58
34	1C179	2019B1	0.77	0.3	0.65	95.58	0.58
35	1C196	2019B2-S1	0.78	0.33	0.61	100	0.61
36	1C205	2020B2-S2	0.78	0.33	0.68	100	0.61
37	1C213	2020B3	0.81	0.27	0.62	100	0.59
38	1C215	2020B4	0.81	0.27	0.68	100	0.59
39	1C218	2020B5	0.82	0.33	0.74	82.8	0.52
40	1C227	2016 Zhong 9-TBM	0.58	0.33	0.73	98.89	0.77
41	1C233	HAU-CR	0.5	0.39	0.71	92.47	0.81
42	1C238	HAU-CR typeIII	0.5	0.27	0.7	97.97	0.75
43	1C242	Huayou-2	0.51	0.33	0.45	100	0.6
44	1C247	H9722	0.47	0.37	0.74	98.96	0.88
45	1C248	H9722 Var1	0.47	0.37	0.78	98.96	0.88
46	1C249	S8R	0.56	0.23	0.43	98.54	0.54
47	1C254	QY211R	0.5	0.39	0.71	92.47	0.81
48	1C259	Ningza1R	0.87	0.3	0.63	71.26	0.23
49	1C264	Q86R	0.47	0.37	0.71	98.96	0.88
50	1C267	SH11	0.82	0.33	0.74	82.8	0.52
51	1C271	SH11za-1	0.82	0.33	0.72	82.8	0.52
52	1C274	Y6-S1	0.69	0.29	0.64	98.96	0.64
53	1C275	2000-5R-S1	0.87	0.3	0.63	71.26	0.23
54	1C276	HYZ01R	0.52	0.29	0.53	100	0.6

55	1C281	Zhong 821R-S1	0.69	0.29	0.64	98.96	0.64
56	1C282	Z6C Var1	0.69	0.29	0.64	98.96	0.64
57	1C283	Z6C-S1	0.6	0.39	0.51	98.82	0.69
58	1C284	QSC-S1	0.47	0.37	0.76	98.96	0.88
59	1C285	QSC-S2	0.47	0.37	0.72	98.96	0.88
60	1C287	S9R	0.58	0.33	0.71	98.89	0.77
61	1C288	CY1R	0.86	0.37	0.37	84.73	0.31
62	1C290	S11R-S1	0.52	0.29	0.43	100	0.6
63	1C293	Liu4	0.81	0.27	0.68	100	0.59
64	1C294	Q7C	0.78	0.33	0.61	100	0.61
65	1C297	Q7C-Var1	0.78	0.33	0.59	100	0.61
66	1C298	Q10C-S1	0.88	0.23	0.65	95.69	0.5
67	1C300	Q10C-S2	0.42	0.34	0.63	99	0.82
68	1C301	Q10R	0.42	0.34	0.63	99	0.82
69	1C305	D1526-S1	0.67	0.4	0.74	98.88	0.79
70	1C306	CZL-20A	0.42	0.34	0.63	99	0.82
71	1C309	CZL-2012	0.88	0.23	0.7	95.69	0.5
72	1C311	CZL-20	0.88	0.23	0.7	95.69	0.5
73	1C312	CH15R	0.82	0.33	0.74	82.8	0.52
74	6C10	Zhongshuang 7-S2	0.77	0.3	0.65	95.58	0.58
75	6C21	Zhongshuang 9S4	0.52	0.29	0.43	100	0.6
76	6C47	Zhongshuang 9II	0.77	0.3	0.65	95.58	0.58
77	6C64	CZ49-S2	0.87	0.3	0.63	71.26	0.23
78	6C70	H15-S2	0.81	0.27	0.68	100	0.59
79	6C76	New B1	0.86	0.37	0.47	84.73	0.31
80	6C88	ZY18-S2	0.82	0.33	0.74	82.8	0.52
81	6C96	2010B1-S2	0.42	0.34	0.63	99	0.82
82	6C100	2010B4	0.47	0.37	0.74	98.96	0.88
83	6C109	2010B7-S2	0.48	0.37	0.58	100	0.78

84	6C124	2012B1-S3	0.52	0.29	0.53	100	0.6
85	6C163	9722	0.5	0.39	0.71	92.47	0.81
86	6C184	Qinyan 211R	0.48	0.37	0.58	100	0.78
87	6C212	Y6-S2	0.69	0.29	0.64	98.96	0.64
88	6C214	2000-5R-S2	0.87	0.3	0.63	71.26	0.23
89	6C216	HYZ01R var1	0.52	0.29	0.63	100	0.6
90	6C226	Zhong 821R-S2	0.75	0.24	0.58	100	0.53
91	6C229	Z6C-S2	0.6	0.39	0.53	98.82	0.69
92	6C230	QSC-S3	0.47	0.37	0.74	98.96	0.88
93	6C239	S11R-S2	0.52	0.29	0.43	100	0.6
94	6C242	Qin7C	0.47	0.37	0.74	98.96	0.88
95	6C251	Q10C Var1	0.88	0.23	0.7	95.69	0.5
96	6C260	D1526-S2	0.67	0.4	0.74	98.88	0.79
97	2E10	Qin 3	0.47	0.37	0.74	98.96	0.88
98	2E11	Westar	0.67	0.4	0.74	98.88	0.79
99	2E12	Bronowski-S1	0.88	0.23	0.72	95.69	0.5
100	2E13	Bronowski-S2	0.88	0.23	0.74	95.69	0.5
101	2E14	Gan 1MP	0.47	0.37	0.74	98.96	0.88
102	2E15	Zhongyou 821var1	0.81	0.27	0.68	100	0.59
103	2E16	C3	0.75	0.24	0.58	100	0.53
104	2E17	KS1701	0.58	0.33	0.71	98.89	0.77
105	2E18	KS2002	0.6	0.39	0.53	98.82	0.69
106	2E19	KS2185	0.86	0.37	0.37	84.73	0.31
107	2E20	KS3017	0.6	0.39	0.53	98.82	0.69
108	2E21	KS3068	0.65	0.21	0.57	89.65	0.47
109	2E22	KS3073	0.67	0.4	0.74	98.88	0.79
110	2E23	KS3074	0.67	0.4	0.74	98.88	0.79
111	2E24	KS3077	0.69	0.29	0.64	98.96	0.64
112	2E25	KS3132	0.69	0.29	0.64	98.96	0.64

113	2E26	KS3248	0.75	0.24	0.58	100	0.53
114	2E27	KS3254	0.77	0.3	0.65	95.58	0.58
115	2E28	KS3302	0.65	0.21	0.57	89.65	0.47
116	2E29	KS3350	0.77	0.3	0.65	95.58	0.58
117	2E30	KS3357	0.78	0.33	0.61	100	0.61
118	2E31	KS4022	0.78	0.33	0.61	100	0.61
119	2E32	New B. napus	0.82	0.33	0.74	82.8	0.52
120	2E33	Viking	0.67	0.4	0.74	98.88	0.79
121	2E34	Profit	0.87	0.3	0.63	71.26	0.23
122	2E35	Elect	0.42	0.34	0.63	99	0.82
123	2E36	SP Arama	0.65	0.21	0.57	89.65	0.47
124	2E37	82089	0.51	0.33	0.47	100	0.6
125	2E38	Jetton	0.56	0.23	0.46	98.54	0.54
126	2E39	Marathow	0.81	0.27	0.68	100	0.59
127	2E40	Runba Var1	0.51	0.33	0.46	100	0.6
128	2E41	Runba	0.5	0.27	0.7	97.97	0.75
129	2E42	Shaan 2C	0.6	0.39	0.53	98.82	0.69
130	2E43	Zhongyou 821	0.81	0.27	0.68	100	0.59
131	2E44	Midas	0.82	0.33	0.74	82.8	0.52

Table S6. Main agronomic traits of 131 *B. napus* accessions.

No.	Code	Name	PH (cm)	SPFDPB (cm)	NB (cm)	LTM (cm)	NSTR	NSP	NS	TSW (g)	SYP (g)
1	1C04	HAU B-gan	175.20	53.00	9.60	57.80	76.00	314.00	21.58	3.28	15.99
2	1C08	Zhongshuang 2S1	191.00	69.20	9.80	62.80	84.00	483.60	20.80	3.92	34.44
3	1C15	Zhongshuang 2S2	179.00	83.40	6.80	60.20	66.40	355.60	13.40	3.51	12.27
4	1C19	Zhongshuang 7-S1	181.40	80.60	6.80	61.40	68.60	271.00	21.08	3.77	16.30
5	1C23	Zhongshuang 5	109.20	0.80	11.10	68.20	48.72	209.90	17.63	2.43	12.38
6	1C25	Zhongshuang 9S1	174.10	50.40	9.00	50.80	65.76	219.10	23.11	2.22	25.07
7	1C27	Zhongshuang 9S2	116.60	1.00	7.50	54.20	33.36	173.20	23.14	2.42	14.25
8	1C29	Zhongshuang 9S3	176.00	62.80	8.80	55.40	72.80	325.20	24.60	3.83	20.79
9	1C35	Zhongshuang 9III-1	180.00	63.00	6.60	61.00	56.40	326.80	18.68	3.56	11.05
10	1C37	Zhongshuang 9III-2	173.50	54.25	10.00	51.00	61.50	342.00	24.50	3.19	14.96
11	1C39	Zhongshuang 4	175.60	73.20	7.80	57.20	77.40	274.20	22.60	3.71	20.98
12	1C41	CZ49-S1	157.00	40.00	8.40	56.40	51.60	307.60	19.98	3.63	14.22
13	1C43	H15-S1	161.80	67.00	6.80	51.80	57.40	240.60	22.68	4.24	15.89
14	1C47	ZS72	184.60	79.20	6.80	56.80	84.80	346.00	25.76	4.54	24.28
15	1C49	ZY18-S1	192.20	96.80	6.80	68.20	93.60	229.40	19.60	4.25	16.76
16	1C51	Shaan 2B	176.20	72.20	9.80	50.60	77.40	428.60	18.16	3.52	19.25
17	1C53	2010B1-S1	176.80	58.40	8.80	63.60	88.00	600.00	17.66	3.70	28.13
18	1C55	2010B6	160.10	51.70	8.20	52.20	52.90	200.70	21.68	2.00	11.56
19	1C57	2010B7-S1	164.90	50.30	8.40	57.50	62.71	206.30	20.44	2.12	17.69
20	1C61	2011B2-S1	166.30	63.10	9.40	47.00	61.53	277.50	22.54	2.04	21.40
21	1C65	2011B2-S2	166.30	60.80	8.20	55.20	67.75	196.00	18.93	2.66	14.67
22	1C67	2012B1-S1	173.20	54.30	12.50	43.60	46.39	350.00	18.44	2.03	15.06
23	1C69	2012B1-S2	166.00	71.80	8.40	52.00	76.40	309.20	19.64	3.06	14.63
24	1C71	2012B3	159.80	71.00	8.60	42.20	74.00	392.80	21.84	3.78	24.96

25	1C77	2016B1	157.30	43.90	12.00	46.00	72.36	304.80	13.83	2.91	16.20
26	1C84	2017B2	187.80	73.80	8.40	59.20	81.20	309.00	20.72	5.18	20.29
27	1C90	2017B4	173.80	75.00	9.00	45.60	62.40	272.40	20.92	4.08	19.00
28	1C95	2018B1	204.60	108.80	8.40	44.60	66.40	354.60	14.60	3.37	7.08
29	1C102	2018B2	167.80	57.80	7.40	61.20	68.20	265.80	18.78	4.11	15.47
30	1C120	2018B3	164.20	64.80	6.00	48.00	50.20	211.60	24.20	3.17	11.75
31	1C131	2018B4	162.20	64.60	8.00	55.80	76.40	333.60	20.60	3.54	19.67
32	1C138	2018B8	177.20	72.40	7.20	51.40	55.40	270.60	14.64	3.43	7.15
33	1C167	2018B9	199.60	89.40	9.00	52.00	71.20	412.20	18.48	2.95	21.40
34	1C179	2019B1	182.20	78.00	7.40	52.60	49.20	236.40	14.76	3.35	8.99
35	1C196	2019B2-S1	164.40	55.20	7.20	67.60	69.60	269.40	20.40	4.37	13.89
36	1C205	2020B2-S2	161.80	68.00	5.80	53.80	63.80	240.80	16.64	3.67	9.27
37	1C213	2020B3	141.50	28.70	10.10	50.10	54.12	170.00	24.40	2.70	38.25
38	1C215	2020B4	156.30	53.60	8.50	44.80	55.05	174.10	21.76	2.29	21.87
39	1C218	2020B5	157.00	57.10	9.40	47.60	61.57	203.50	19.22	2.38	16.12
40	1C227	2016 Zhong 9-TBM	151.80	42.80	9.50	46.50	52.64	170.60	24.68	1.97	22.00
41	1C233	HAU-CR	146.20	48.50	9.50	49.10	55.42	167.10	24.39	1.79	16.71
42	1C238	HAU-CR typeIII	163.70	61.50	8.00	54.60	59.19	237.60	17.36	2.32	12.31
43	1C242	Huayou-2	158.20	38.00	10.20	50.30	58.90	299.40	21.16	2.25	24.87
44	1C247	H9722	108.10	15.80	9.40	46.70	39.62	223.40	18.97	2.39	15.84
45	1C248	H9722 Var1	108.10	15.80	9.40	46.70	39.62	223.40	18.97	2.39	15.84
46	1C249	S8R	109.20	0.80	11.10	68.20	48.72	209.90	17.63	2.43	12.38
47	1C254	QY211R	116.60	1.00	7.50	54.20	33.36	173.20	23.14	2.42	14.25
48	1C259	Ningza1R	116.60	1.00	7.50	54.20	33.36	173.20	23.14	2.42	14.25
49	1C264	Q86R	141.50	28.70	10.10	50.10	54.12	170.00	24.40	2.70	38.25
50	1C267	SH11	146.20	48.50	9.50	49.10	55.42	167.10	24.39	1.79	16.71

51	1C271	SH11za-1	151.80	42.80	9.50	46.50	52.64	170.60	24.68	1.97	22.00
52	1C274	Y6-S1	156.30	53.60	8.50	44.80	55.05	174.10	21.76	2.29	21.87
53	1C275	2000-5R-S1	157.00	40.00	8.40	56.40	51.60	307.60	19.98	3.63	14.22
54	1C276	HYZ01R	157.00	57.10	9.40	47.60	61.57	203.50	19.22	2.38	16.12
55	1C281	Zhong 821R-S1	157.00	40.00	8.40	56.40	51.60	307.60	19.98	3.63	14.22
56	1C282	Z6C Var1	157.00	40.00	8.40	56.40	51.60	307.60	19.98	3.63	14.22
57	1C283	Z6C-S1	157.30	43.90	12.00	46.00	72.36	304.80	13.83	2.91	16.20
58	1C284	QSC-S1	158.20	38.00	10.20	50.30	58.90	299.40	21.16	2.25	24.87
59	1C285	QSC-S2	159.80	71.00	8.60	42.20	74.00	392.80	21.84	3.78	24.96
60	1C287	S9R	160.10	51.70	8.20	52.20	52.90	200.70	21.68	2.00	11.56
61	1C288	CY1R	161.80	67.00	6.80	51.80	57.40	240.60	22.68	4.24	15.89
62	1C290	S11R-S1	161.80	68.00	5.80	53.80	63.80	240.80	16.64	3.67	9.27
63	1C293	Liu4	161.80	67.00	6.80	51.80	57.40	240.60	22.68	4.24	15.89
64	1C294	Q7C	162.20	64.60	8.00	55.80	76.40	333.60	20.60	3.54	19.67
65	1C297	Q7C-Var1	163.70	61.50	8.00	54.60	59.19	237.60	17.36	2.32	12.31
66	1C298	Q10C-S1	164.20	64.80	6.00	48.00	50.20	211.60	24.20	3.17	11.75
67	1C300	Q10C-S2	164.40	55.20	7.20	67.60	69.60	269.40	20.40	4.37	13.89
68	1C301	Q10R	164.90	50.30	8.40	57.50	62.71	206.30	20.44	2.12	17.69
69	1C305	D1526-S1	164.90	50.30	8.40	57.50	62.71	206.30	20.44	2.12	17.69
70	1C306	CZL-20A	166.00	71.80	8.40	52.00	76.40	309.20	19.64	3.06	14.63
71	1C309	CZL-2012	166.00	71.80	8.40	52.00	76.40	309.20	19.64	3.06	14.63
72	1C311	CZL-20	166.30	63.10	9.40	47.00	61.53	277.50	22.54	2.04	21.40
73	1C312	CH15R	166.30	60.80	8.20	55.20	67.75	196.00	18.93	2.66	14.67
74	6C10	Zhongshuang 7-S2	181.40	80.60	6.80	61.40	68.60	271.00	21.08	3.77	16.30
75	6C21	Zhongshuang 9S4	116.60	1.00	7.50	54.20	33.36	173.20	23.14	2.42	14.25
76	6C47	Zhongshuang 9II	173.50	54.25	10.00	51.00	61.50	342.00	24.50	3.19	14.96

77	6C64	CZ49-S2	157.00	40.00	8.40	56.40	51.60	307.60	19.98	3.63	14.22
78	6C70	H15-S2	161.80	67.00	6.80	51.80	57.40	240.60	22.68	4.24	15.89
79	6C76	New B1	167.80	57.80	7.40	61.20	68.20	265.80	18.78	4.11	15.47
80	6C88	ZY18-S2	192.20	96.80	6.80	68.20	93.60	229.40	19.60	4.25	16.76
81	6C96	2010B1-S2	176.80	58.40	8.80	63.60	88.00	600.00	17.66	3.70	28.13
82	6C100	2010B4	173.20	54.30	12.50	43.60	46.39	350.00	18.44	2.03	15.06
83	6C109	2010B7-S2	164.90	50.30	8.40	57.50	62.71	206.30	20.44	2.12	17.69
84	6C124	2012B1-S3	166.00	71.80	8.40	52.00	76.40	309.20	19.64	3.06	14.63
85	6C163	9722	173.50	54.25	10.00	51.00	61.50	342.00	24.50	3.19	14.96
86	6C184	Qinyan 211R	173.50	54.25	10.00	51.00	61.50	342.00	24.50	3.19	14.96
87	6C212	Y6-S2	173.80	75.00	9.00	45.60	62.40	272.40	20.92	4.08	19.00
88	6C214	2000-5R-S2	174.10	50.40	9.00	50.80	65.76	219.10	23.11	2.22	25.07
89	6C216	HYZ01R var1	175.20	53.00	9.60	57.80	76.00	314.00	21.58	3.28	15.99
90	6C226	Zhong 821R-S2	175.60	73.20	7.80	57.20	77.40	274.20	22.60	3.71	20.98
91	6C229	Z6C-S2	176.00	62.80	8.80	55.40	72.80	325.20	24.60	3.83	20.79
92	6C230	QSC-S3	176.20	72.20	9.80	50.60	77.40	428.60	18.16	3.52	19.25
93	6C239	S11R-S2	176.80	58.40	8.80	63.60	88.00	600.00	17.66	3.70	28.13
94	6C242	Qin7C	176.80	58.40	8.80	63.60	88.00	600.00	17.66	3.70	28.13
95	6C251	Q10C Var1	177.20	72.40	7.20	51.40	55.40	270.60	14.64	3.43	7.15
96	6C260	D1526-S2	179.00	83.40	6.80	60.20	66.40	355.60	13.40	3.51	12.27
97	2E10	Qin 3	180.00	63.00	6.60	61.00	56.40	326.80	18.68	3.56	11.05
98	2E11	Westar	181.40	80.60	6.80	61.40	68.60	271.00	21.08	3.77	16.30
99	2E12	Bronowski-S1	181.40	80.60	6.80	61.40	68.60	271.00	21.08	3.77	16.30
100	2E13	Bronowski-S2	182.20	78.00	7.40	52.60	49.20	236.40	14.76	3.35	8.99
101	2E14	Gan 1MP	184.60	79.20	6.80	56.80	84.80	346.00	25.76	4.54	24.28
102	2E15	Zhongyou 821var1	187.80	73.80	8.40	59.20	81.20	309.00	20.72	5.18	20.29

103	2E16	C3	191.00	69.20	9.80	62.80	84.00	483.60	20.80	3.92	34.44
104	2E17	KS1701	157.00	40.00	8.40	56.40	51.60	307.60	19.98	3.63	14.22
105	2E18	KS2002	192.20	96.80	6.80	68.20	93.60	229.40	19.60	4.25	16.76
106	2E19	KS2185	192.20	96.80	6.80	68.20	93.60	229.40	19.60	4.25	16.76
107	2E20	KS3017	199.60	89.40	9.00	52.00	71.20	412.20	18.48	2.95	21.40
108	2E21	KS3068	204.60	108.80	8.40	44.60	66.40	354.60	14.60	3.37	7.08
109	2E22	KS3073	108.10	15.80	9.40	46.70	39.62	223.40	18.97	2.39	15.84
110	2E23	KS3074	109.20	0.80	11.10	68.20	48.72	209.90	17.63	2.43	12.38
111	2E24	KS3077	116.60	1.00	7.50	54.20	33.36	173.20	23.14	2.42	14.25
112	2E25	KS3132	116.60	1.00	7.50	54.20	33.36	173.20	23.14	2.42	14.25
113	2E26	KS3248	141.50	28.70	10.10	50.10	54.12	170.00	24.40	2.70	38.25
114	2E27	KS3254	146.20	48.50	9.50	49.10	55.42	167.10	24.39	1.79	16.71
115	2E28	KS3302	151.80	42.80	9.50	46.50	52.64	170.60	24.68	1.97	22.00
116	2E29	KS3350	156.30	53.60	8.50	44.80	55.05	174.10	21.76	2.29	21.87
117	2E30	KS3357	157.00	40.00	8.40	56.40	51.60	307.60	19.98	3.63	14.22
118	2E31	KS4022	157.00	57.10	9.40	47.60	61.57	203.50	19.22	2.38	16.12
119	2E32	New B. napus	157.00	40.00	8.40	56.40	51.60	307.60	19.98	3.63	14.22
120	2E33	Viking	157.00	40.00	8.40	56.40	51.60	307.60	19.98	3.63	14.22
121	2E34	Profit	157.30	43.90	12.00	46.00	72.36	304.80	13.83	2.91	16.20
122	2E35	Elect	158.20	38.00	10.20	50.30	58.90	299.40	21.16	2.25	24.87
123	2E36	SP Arama	159.80	71.00	8.60	42.20	74.00	392.80	21.84	3.78	24.96
124	2E37	82089	160.10	51.70	8.20	52.20	52.90	200.70	21.68	2.00	11.56
125	2E38	Jetton	161.80	67.00	6.80	51.80	57.40	240.60	22.68	4.24	15.89
126	2E39	Marathow	161.80	68.00	5.80	53.80	63.80	240.80	16.64	3.67	9.27
127	2E40	Runba Var1	161.80	67.00	6.80	51.80	57.40	240.60	22.68	4.24	15.89
128	2E41	Runba	162.20	64.60	8.00	55.80	76.40	333.60	20.60	3.54	19.67

129	2E42	Shaan 2C	163.70	61.50	8.00	54.60	59.19	237.60	17.36	2.32	12.31
130	2E43	Zhongyou 821	164.20	64.80	6.00	48.00	50.20	211.60	24.20	3.17	11.75
131	2E44	Midas	164.40	55.20	7.20	67.60	69.60	269.40	20.40	4.37	13.89

Note: Plant height (PH), branching height (BH), number of primary branches per plant (NB), Length of the terminal raceme (LTM), number of siliques on the terminal raceme (NSTR), number of siliques per plant (NSP), number of seeds per silique (NS), thousand-seed weight (TSW), seed yield per plant (SYP).
